# Real-time fluorescent monitoring of phase I xenobiotic-metabolizing enzymes

**DOI:** 10.1039/d4ra00127c

**Published:** 2024-03-15

**Authors:** Hajra Iqbal, Kainat Ilyas, Muhammad Sajid Hamid Akash, Kanwal Rehman, Amjad Hussain, Jamshed Iqbal

**Affiliations:** a Department of Pharmaceutical Chemistry, Government College University Faisalabad Pakistan sajidakash@gcuf.edu.pk; b Department of Pharmacy, The Women University Multan Pakistan kanwalrehman@wum.edu.pk; c Institute of Chemistry, University of Okara Okara Pakistan; d Centre for Advanced Drug Research, COMSATS University Islamabad, Abbottabad Campus Abbottabad 22044 Pakistan

## Abstract

This article explores the intricate landscape of advanced fluorescent probes crafted for the detection and real-time monitoring of phase I xenobiotic-metabolizing enzymes. Employing state-of-the-art technologies, such as fluorescence resonance energy transfer, intramolecular charge transfer, and solid-state luminescence enhancement, this article unfolds a multifaceted approach to unraveling the dynamics of enzymatic processes within living systems. This encompassing study involves the development and application of a diverse range of fluorescent probes, each intricately designed with tailored mechanisms to heighten sensitivity, providing dynamic insights into phase I xenobiotic-metabolizing enzymes. Understanding the role of phase I xenobiotic-metabolizing enzymes in these pathophysiological processes, is essential for both medical research and clinical practice. This knowledge can guide the development of approaches to prevent, diagnose, and treat a broad spectrum of diseases and conditions. This adaptability underscores their potential clinical applications in cancer diagnosis and personalized medicine. Noteworthy are the trifunctional fluorogenic probes, uniquely designed not only for fluorescence-based cellular imaging but also for the isolation of cellular glycosidases. This innovative feature opens novel avenues for comprehensive studies in enzyme biology, paving the way for potential therapeutic interventions. The research accentuates the selectivity and specificity of the probes, showcasing their proficiency in distinguishing various enzymes and their isoforms. The sophisticated design and successful deployment of these fluorescent probes mark significant advancements in enzymology, providing powerful tools for both researchers and clinicians. Beyond their immediate applications, these probes offer illuminating insights into disease mechanisms, facilitating early detection, and catalyzing the development of targeted therapeutic interventions. This work represents a substantial leap forward in the field, promising transformative implications for understanding and addressing complex biological processes. In essence, this research heralds a new era in the development of fluorescent probes, presenting a comprehensive and innovative approach that not only expands the understanding of cellular enzyme activities but also holds great promise for practical applications in clinical settings and therapeutic endeavors.

## Abstract 2

Applications of chemosensors of phase I metabolizing enzymes.

## Graphical abstract

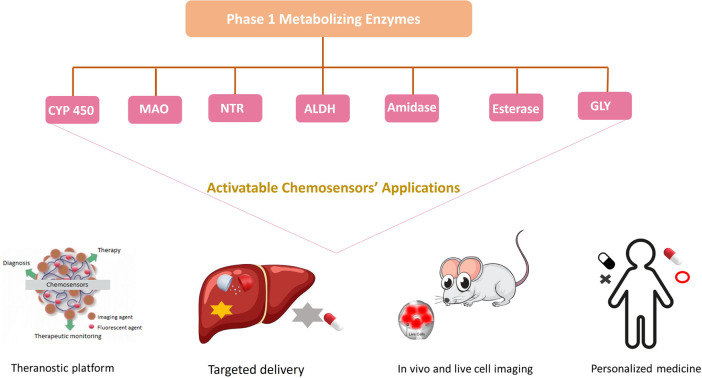

## 1. Introduction

Chemosensors serve a pivotal role in the real-time detection and monitoring of enzymatic reactions and the diffusion of analytes through biological membranes. Specifically referred to as enzyme-directed chemosensors, these instruments generate an optical signal, predominantly in the form of fluorescence, upon interaction with exogenous chemicals.^1^ Human exposure to these chemicals, commonly known as xenobiotics, is continuous. While generally harmless at lower concentrations, they pose potential lethality at higher concentrations.^2^ The toxicity of xenobiotics is contingent upon the physiological interplay between storage conditions and the site specificity of their metabolites within the human body.^3,4^ Within the human body, various enzymes are intricately involved in the metabolism and excretion of xenobiotics, encompassing drugs, pollutants, cosmetics, and diverse nutrients.^5^ Exogenous xenobiotics present a substantial risk to both human health and the environment due to their carcinogenicity, propensity for accumulation, and inherent toxicity.^6^ Prolonged exposure to these substances at elevated concentrations may result in chronic abnormalities, including but not limited to growth retardation, immunological and neurological impairments, learning difficulties, reproductive problems, and the manifestation of various human diseases such as Parkinson's and Alzheimer's.^7,8^ Mitigating the acute and chronic toxicity associated with exogenous xenobiotics is attainable through leveraging the natural detoxification mechanisms inherent in the human body.^9^

**Fig. 1:**
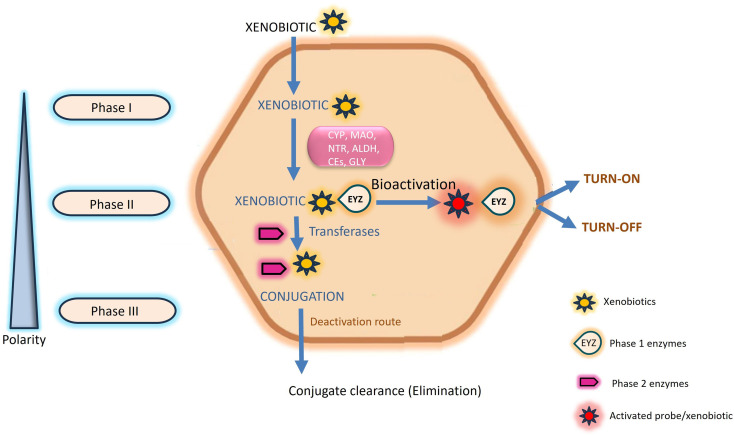
Schematic representation of the chemical transformation of xenobiotics. Phase I enzymes are symbolized by the term “bubble”. After interacting with enzymes, probes either turn-on or turn-off which indicate the pathological conditions.

This natural detoxification mechanism facilitating the elimination and excretion of diverse xenobiotics is systematically categorized into three phases, denoted as Phase I, II, and III, as illustrated in Fig. 1.^10^ Phase I metabolism entails the modification and biotransformation of xenobiotics and toxic substances by introducing or unmasking polar groups such as –SH, –OH, –OR, and –NH_2_ into the parent compound. This intricate biotransformation process is orchestrated by a complex system of enzymes.^3,11^ In Phase II, drug-metabolizing enzymes, notably transferases, orchestrate conjugation reactions. These enzymes catalyze the conjugation of Phase I reaction products with compounds such as glucuronic acid, glutathione, and sulfate.^10,12^ Phase III, termed the extrusion phase, further enhances the polarity of these metabolites through hydroxylation, rendering them more water-soluble. Subsequently, these hydroxylated metabolites are primed for excretion by the liver or kidneys, as depicted in Fig. 1.^3,13^ The enzymes involved in transportation and conjugation play a crucial role in absorption, distribution, metabolism, and excretion processes. Any aberration in their function may result in alterations to the pharmacokinetics and pharmacodynamics of drugs.^9,14^

Phase I enzymes have garnered attention in the development of enzyme-directed fluorescence chemosensors, owing to their inclusion of pharmacologically significant enzymes such as esterase, cytochrome P450 family, monoamine oxidase, nitroreductase, aldehyde dehydrogenase, and glycosidase.^10,15^ These enzymes actively participate in the metabolism and activation of numerous prodrugs, with their overexpression in cancer cells marking them as potential biomarkers for early-stage cancer diagnosis and monitoring therapeutic efficacy of cancer drugs.^16–19^ Anticipating a surge in cancer cases, the World Health Organization predicts 24 million new cases and 14.4 million annual deaths by 2035. Early and accurate diagnosis, crucial for improving survival rates, hinges on detecting aberrant enzyme activity, which is directly linked to malignancies. The exact localization of these enzymes in live cancer cells is imperative for early cancer diagnosis and treatment assessment.^16^ Traditional methods like high-performance liquid chromatography (HPLC) and mass spectrometry (MS) fall short in providing real-time monitoring of Phase I metabolizing enzymes.^20^ In contrast, fluorescence imaging, offering high spatiotemporal resolution, emerges as an effective approach for safe, rapid, and sensitive detection of enzyme activity in cells, tissues, organs, and animals.^20,21^ Consequently, there is a pressing need for the development of enhanced fluorescent chemosensors designed to selectively detect Phase I xenobiotic metabolizing enzymes. This demand arises from critical research domains including theranostics, targeted drug design, and the visual detection of drug resistance.^10^

This comprehensive review delves into recent advancements in fluorescent chemosensors, focusing on their diagnostic applications for Phase I enzymes based on enzymatic oxidative, reductive, and hydrolytic reactions. The review highlights *in vitro* and *in vivo* diagnostic uses of small molecule-based fluorescent chemosensors for Phase I xenobiotic metabolizing enzymes, exploring how xenobiotic metabolism induces changes in the fluorescence of small molecules through structural modifications. Various advantages of employing xenobiotic chemosensors for theranostic purposes and the diverse applications of xenobiotic-metabolizing enzymes in biomedicine are thoroughly considered. Emphasis is placed on the efficacy of small-molecule fluorescent probes for diagnosing cancer and assessing resistance to anti-cancer drugs, with a focus on forthcoming avenues in designing and developing small molecular fluorescent probes activated by enzymes for real-time detection of Phase I metabolic enzymes.

## 2. Comprehensive scheme

During the past decade there has been a significant focus on fluorescent chemosensors within the realm of supramolecular chemistry.^22^ These sensors have garnered considerable interest due to their high selectivity and sensitivity in fluorescent assays.^23^ In the development of innovative fluorescent chemosensors, ongoing exploration of novel sensing mechanisms that link recognition and signal reporting elements continues to be a focal point of interest. This general scheme highlights the interplay between fluorescent probes and Phase I enzymes, where the enzymatic transformations trigger changes in fluorescence intensity. The specific design and mechanism may vary based on the nature of the probe and the targeted Phase I enzyme. Chemoresponsive sensing occupies a pivotal role in its capacity for selective and real-time detection of specific chemical substances.^24^ Chemoresponsive sensing is given priority than other sensing mechanism because these sensors offer precision by specifically recognizing biomarkers associated with various diseases.^25^ Their selectivity ensures targeted responses to molecules, facilitating precise diagnostics. With real-time monitoring capabilities, chemoresponsive sensing becomes invaluable in situations where timely detection of biomarkers is critical for early diagnosis and effective treatment.^26^ Phase I enzymes involve a mechanism where the probes undergo specific enzymatic transformations leading to changes in fluorescence intensity.^27^ The fluorescent probe is designed with a fluorophore and a quencher in proximity, leading to quenched fluorescence in the native state. The probe interacts with Phase I enzymes by accessing their active site, initiating a specific catalytic transformation involving oxidation, reduction, or hydrolysis. This enzymatic activity induces structural changes in the probe, impacting its fluorophore-quencher arrangement. The liberation of the fluorophore from the quencher marks a crucial transition, leading to heightened fluorescence in the activated state. This increased fluorescence serves as a distinctive signal, indicating the presence and activity of Phase I enzymes, facilitating efficient detection and imaging. In certain designs, the enzymatic transformation may lead to a quenched state due to conformational changes or interactions that reduce fluorescence.^28^

Photophysical processes such as Intramolecular Charge Transfer, Förster Resonance Energy Transfer (FRET), Photoinduced Electron Transfer (PET), and Excited-State Intramolecular Proton Transfer (ESIPT) play crucial roles in the design and functioning of fluorescent probes. Intramolecular Charge Transfer is a phenomenon that involves the redistribution of electron density within a molecule upon excitation, causing changes in its electronic structure. Probes designed with ICT properties demonstrate altered fluorescence in response to variations in the local environment, such as changes in polarity or solvent conditions. These probes are valuable for sensing and imaging applications, particularly in environments where specific molecular conditions need to be monitored.^29^ Förster Resonance Energy Transfer (FRET) facilitates energy transfer between a donor and an acceptor fluorophore in close proximity, resulting in either quenching or enhancement of fluorescence.^30^ Probes incorporating FRET capabilities are employed for detecting molecular interactions, conformational changes, or proximity to specific targets. Photoinduced Electron Transfer (PET) involves the transfer of an electron from one molecular entity to another upon excitation, influencing the fluorescence properties. Probes with PET characteristics can be tailored to respond to specific analytes or environmental changes, making them versatile tools for sensing various chemical and biological entities, including ions and reactive oxygen species.^31^ Excited-State Intramolecular Proton Transfer (ESIPT) is characterized by the transfer of a proton within a molecule in the excited state, leading to alterations in fluorescence properties. Probes designed with ESIPT properties are particularly useful for pH sensing and imaging applications. These probes find applications in monitoring pH changes within biological systems, offering insights into cellular environments and their dynamic nature. Understanding and manipulating these processes allows for the development of highly sensitive and selective fluorescent probes tailored for diverse analytical and biological purposes.^32^

## 3. Phase I xenobiotic-metabolizing enzymes

### 3.1 Chemosensors responsive to cytochrome P450

**Fig. 2:**
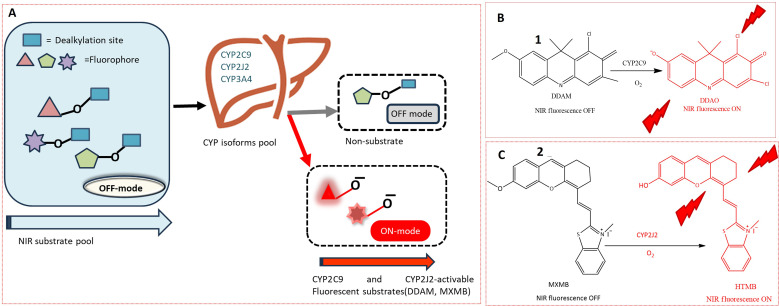
(A) *In vitro* screening of near-infrared fluorescent substrates for CYP2C9 and CYP2J2, using an activity-based approach. (B) Fluorescence response of 1 towards CYP2C9. (C) Fluorogenic reaction of 2 toward SCYP2J2. 1 and 2 exhibits increase in fluorescence which is depicted by red colour in figure.

The cytochrome P450 (CYP) family, a heme-containing superfamily, assumes a pivotal role in the Phase I metabolism of diverse endogenous compounds, drugs, and xenobiotics.^33,34^ As quintessential biocatalysts, CYPs exhibit the capability to catalyze a wide range of reactions across various substrate structures.^3,35,36^ Under mild conditions, CYPs can facilitate stereo-selective oxidation of inactive C–H bonds in organic molecules.^37,38^ Studies have established a strong association between CYPs and various diseases.^36,39^ Consequently, the detection of these enzymes using fluorogenic agents has become a significant area of interest.^40^ However, the development of fluorescent probes for CYPs encounters a major obstacle due to the homologous protein structure, resulting in similarities in catalytic activity and fluorescence emission spectra among various CYP isoforms. Despite this challenge, several probes have been successfully designed for the substrate-specific detection of different CYP450 isoforms. A novel double-filtering approach was employed based on the structure specificity and catalytic activity of human CYP isoforms. The screening involved a two-step process, starting with dealkylation reactions using a series of near-infrared (NIR) fluorophores. Subsequently, CYP-dependent *in vitro* screening of these fluorescent probes was conducted, leading to the construction of an activated CYPs NIR fluorescent probes pool. This screening involved the use of reverse protein-ligand docking between human liver microsomes and fluorescent substrates, as illustrated in Fig. 2A. Through a combination of real and virtual screening, two fluorescent probes (1 and 2) were specifically designed for the detection of CYP2C9 and CYP2J2, respectively. The maximum fluorescence emission spectrum of probe 1 was observed at 658 nm, as depicted in Fig. 2B. This real-time tracking approach was employed for assessing drug–drug interactions, while probe 2 was utilized for detecting CYP2J2, a biomarker for cancer diagnosis and treatment, as shown in Fig. 2C. The potential activity of fluorescent probe 2 in detecting endogenous substances was demonstrated in HeLa and HepG2 cells, with a fluorescence emission spectrum observed in the range of 690–750 nm.^41^

**Fig. 3:**
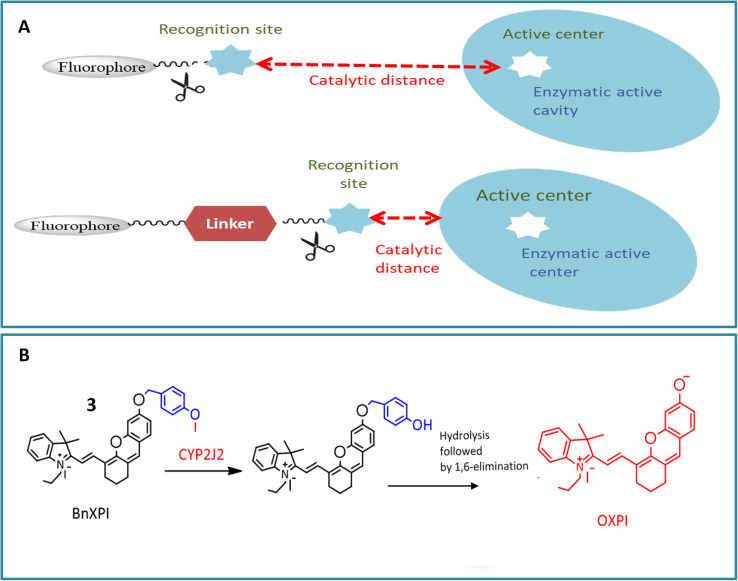
(A) A design approach that utilizes a self-immolative linker to enhance the catalytic efficiency of CYP2J2. The optimization of the probe involves fine-tuning the catalytic distance and recognition moiety. (B) Increase in fluorescence in 3 shown by red color after interacting with CYP2J2.

Another NIR fluorescent probe 3 was designed for real-time tracking of CYP2J2 in complex biological systems, leveraging the overexpression of CYP2J2 in various cancer cells. The basic fluorophore component, (*E*)-2-(2-(6-hydroxy-2,3-dihydro-1*H*-xanthen-4-yl) vinyl)-3,3-dimethyl-1-propyl-3*H*-indol-1-ium iodide (HXPI), was selected for its long aromatic structure, photoemissive properties, and high tissue penetration. A self-immolative linker (*p*-hydroxybenzyl group) was introduced to enhance the catalytic activity of dealkylation by reducing the spatial distance between the catalytic region of CYP2J2 and the metabolic recognition moiety (catalytic distance) as depicted in Fig. 3A. The catalytic activity of CYP2J2 can be improved by decreasing the distance between its heme catalytic unit and *O*-alkyl group, which serves as the metabolic recognition moiety. Introducing a ‘best-fit’ fluorescent probe, especially a *p*-hydroxybenzyl group, facilitates effective interaction between the metabolic recognition site and the catalytic activity of CYP2J2 by reducing the catalytic distance. The isoform-selectivity of the fluorescence chemosensor toward CYP2J2 was improved by optimizing the metabolic recognition moiety (*O*-alkyl group). The *p*-hydroxybenzyl ether derivatives exhibited higher fluorescence signals than *O*-alkylated derivatives. The introduction of the *p*-hydroxybenzyl ether moiety enhanced the catalytic activity of CYP2J2 toward HXPI, leading to a significant fluorescence turn-on response. The validity of probe 3 was confirmed in the presence of CYP2J2, showing a significant fluorescence signal at 718 nm. The selectivity and sensitivity of probe 3 were tested in various carcinoma cell lines, indicating a 46-fold higher activity rate than other isoforms. The probe's efficacy in diagnosing hematological malignancies and their treatment was suggested based on observations in human umbilical vein endothelial cells (HUVEC) showing a strong fluorescence signal.^20^

**Fig. 4:**
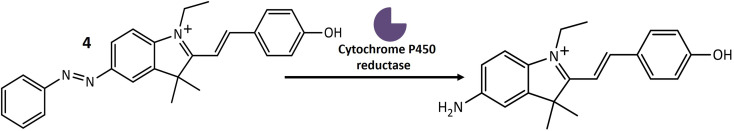
Structure of 4 and the response of 4 towards cytochrome P450 reductase (CPR).

Probe 4 was developed to identify cytochrome P450 reductase (CPR) in cancer cells for hypoxia imaging. CPR is responsible for detoxifying xenobiotics, and probe 4 containing an azo moiety exhibited fluorescence emission at 610 nm in PBS. Probe 4, designed by conjugating an azo group with an electron-withdrawing indole group, demonstrated enhanced sensitivity and selectivity toward CPR under low oxygen partial pressure due to the presence of an azo bond as shown in Fig. 4. The anti-interference effect was evaluated by treating probe 4 with bio thiols, reductants, and inorganic salts, displaying strong fluorescence even at low concentrations of these reductants. Probe 4 showed 11 times stronger fluorescence intensity in a hypoxic environment compared to normoxic conditions. Although the probe revealed that CPR is not the sole reductase capable of reducing the azo bond, it was successfully used for imaging in A549 cells, a human lung cancer cell line. The fluorescence of 4 changed upon interaction with CYP450 reductase and NADH. Probe 4 demonstrated significant optical changes and sensitivity toward cytochrome P450 reductase due to the reduction of the azo bond. The probe exhibited a gradual change in intensity based on concentration and was evaluated for hypoxic imaging of cancer cells with A549 cell lines in the presence of an inhibitor diphenyliodonium chloride (100 μM), showing a weak fluorescence signal even in the presence of the inhibitor. These findings demonstrated the potential ability of the probe to sensitively discriminate oxygen concentration.^42^

**Fig. 5:**
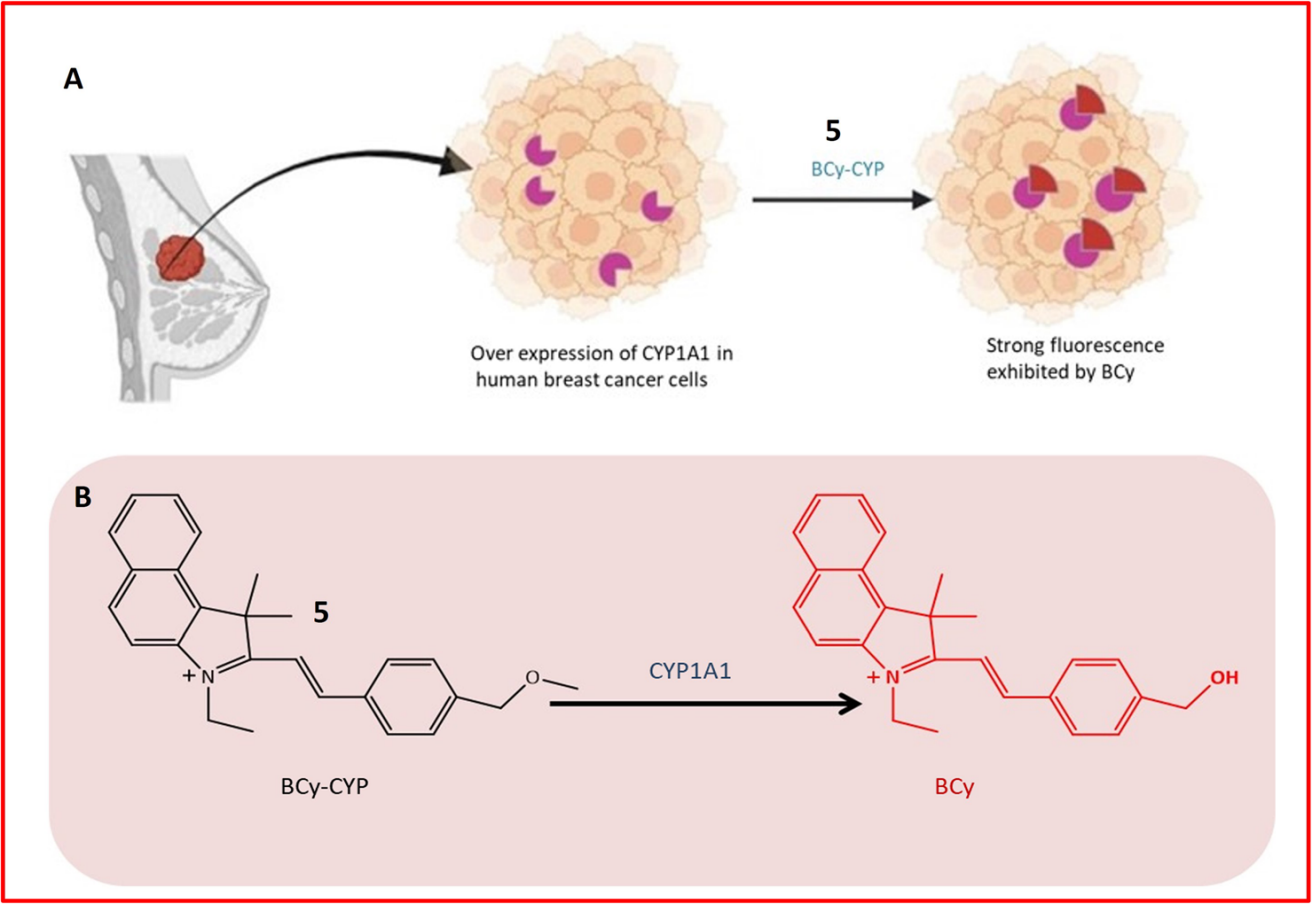
(A) Detection mechanism of BCy-CYP towards CYP1A1 in breast cancer cells. The shape in purple color indicates the overexpression of the enzyme (CYP1A1) in breast tissue while the red color shape indicates increased fluorescence activity of probe upon interaction with the enzyme. (B) Fluorescence response of 5 towards CYP1A1.

Fluorescence probe 5 was designed for the identification of CYP1A1, contributing to the diagnosis and treatment of breast cancer. Elevated expression of CYP1A1 in breast tumors compared to healthy cells was observed in recent studies. Probe 5, an effective small molecule probe, was employed for imaging CYP1A1 in MDA-MB-231 and MCF-7 cells, both in live cells and mouse cells with tumors. The specificity of the probe towards CYP1A1 was examined, and its application for testing CYP1A1 in living cells was assessed. All cells were treated with BCy-CYP at 37 °C, and changes in fluorescence were measured using flow cytometry and laser scanning confocal microscopy. Over the 30 minutes experimental period, the fluorescence of these cells increased progressively. The probe incorporated an ethoxy group as a response unit into the benzoindocyanine (BCy) fluorophore part, displaying fluorescence emission at 630 nm upon dealkylation by CYP1A1 (Table 1). The selectivity of probe 5 was demonstrated on breast cancer cell lines (MDA-MB-231 cells), showing significant fluorescence, and indicating a higher concentration of CYP1A1 in breast cancer cells, as illustrated in Fig. 5A and B. The study also revealed the inhibition of CYP1A1 with carnosol-induced cell death (apoptosis) in breast cancer cells, confirming the synergistic effect of cisplatin and carnosol.^43^

**Table 1:** Comprehensive overview of chemical properties in probes

Enzymes	Probes	Chemical name of probe	Nature of probe	Uses	Ref.
Cytochrome P450	1 and 2	—	Turn-on	Useful for evaluating the risks of drug–drug interactions in clinical settings and for diagnosing tumors	41
	3	*p*-Hydroxybenzyl ether derivatives of (*E*)-2-(2-(6-hydroxy-2,3-dihydro-1*H*-xanthen-4-yl)vinyl)-3,3-dimethyl-1-propyl-3*H*-indol-1-ium iodide	Turn-on	Beneficial for investigating the biological roles of CYP2J2 in the development of tumors	20
	4	Azo group connected to the indole group	Turn-on	Useful in detecting the hypoxic condition of tumor cells	42
	5	Benzoindocyanine (BCy) attached with ethoxy	Turn-on	A valuable marker for the early detection of breast cancer	43
	6	*N*-(3-Carboxy propyl)-4-methoxy-1,8-naphthalimide	Turn-on	A useful tool for exploring the biological functions of CYP1A in toxicological assessments	44
Monoamine oxidases	7 and 8	3-(2-Chlorophenoxy) propan-1-amine unit and propylamine unit	Turn-on	A promising tool for studying MAO-A activity in carcinoma	50
	10	Triarylboron compounds with boron center surrounded by three aromatic groups	Turn-on	Able to distinguish cells that overexpress MAOs from other cell types	52
	11a	Hexyl group connected to the nitrogen atom on the 1,8-naphthalimide	Turn-on	Useful for detecting MAO-A in diverse biological environments	53
	12	Quinoline-malononitrile (QM) with propylamine	Turn-on	Beneficial for promptly detecting MAO-A and MAO-B within living cells	54
Nitroreductase	13	Cyanine structure with an aromatic nitro group	Turn-off	An effective tool for differentiating hypoxic tumors *in vivo*	60
	14	Imidazolering coupled with IR-1048	Turn-on	Useful for hypoxia-activated photothermal therapy and tumor detection	61
	15	Coumarin with nitro-naphthylamide	Turn-on	Beneficial for diagnosing, drug discovery, and treating cancer	62
	16	Naphthylamide core with specific functional groups	Turn-on	Useful for assessing human tumors as a biomarker to evaluate hypoxia	63
	17	Quinoxaline ring	Turn-on	Beneficial for tracking NTR within living cells to diagnose hypoxia in tumors	64
Aldehyde dehydrogenases	18 and 19	BODIPY fluorophore with an amino methyl benzaldehyde moiety	Turn-on	Beneficial for malignant transformation in cancer stem cells	72
	20	Pennsylvania green dye coupled with a benzaldehyde moiety	Turn-on	Helpful for identifying cancer stem cells at the cellular level	73
	21 and 22	Benzoindole-based fundamental structure	Turn-on	An effective tool for detecting cancer stem cells	74
	23	Benzoindole-based fundamental structure with carboxylic, thiol groups and methyl groups	Turn-on	Beneficial for achieving high-contrast imaging of aldehyde dehydrogenase 1A1 in cancer stem cells	75
	24	Acylal group with aromatic aldehyde	Turn-on	Non-invasive imaging ALDH activity in ovarian cancer	76
Esterases	27	2-(2-Hydroxyphenyl) benzothiazole attach with tertiary butyl ester	Turn-on	Beneficial for real-time tracking of endogenous CEs activity in cancer cells drug-induced CEs activity	51
	28	Dihydroxanthene and indole along with cyclohexyl as ring structure	Turn-on	Beneficial for directing esterase activity within mitochondria and differentiating between dead cells and live cells	77
	29	Meso-carboxylate substituted a boron dipyrromethene	Turn on	Clinically useful identifying the pathogenic bacterium *Moraxella catarrhalis* in cultures	85
	30 and 31	6-Amino-1-phenalenone in conjunction with benzoyl (30) and pyruval (31)	Turn-on	Useful for measuring hCES2 activity in tissue samples derived from pancreatic cancer patients	86
	32	Boron-dipyrromethene (BODIPY) based encapsulated within a metal–organic skeleton using zeolite imidazolate framework-8 (ZIF-8)	Turn-off	Useful for monitoring CES1 activity in living cells, aiding in various pathological conditions	87
Fatty acid amide hydrolase (FAAH)	34	7-Amino-3*H*-phenoxazine-3-one	Turn-on	Beneficial for uncovering therapeutic agents for diseases associated with FAAH.	98
Leucine aminopeptidase (LAP)	36	l-Leucine and CHMC-M	Turn-on	Tracking LAP both *in vivo* and *in vitro*, beneficial for diagnosing malignant tumors	105
Gamma-glutamyl transpeptidase (GGT)	37	Terephthalonitrile core with four fluorine atoms attached to aromatic rings, and two glutathione (GSH) molecules	Turn-on	Effectively used for ratiometric imaging of GGT in cancer cells, normal cells, and intricate biological systems	111
Glycosidase	38	2-Hydroxy-5-(1,2,2-triphenylethenyl)-benzaldehyde and 2-aminothiophenol attached with galactose	Turn-on	Assessment of glycosidase activity, beneficial for diagnostic purposes and drug screening assays	120
	39a	Monofluoromethylated attached with β-*N*-acetylhexosaminie	Turn-on	Enable investigations into the function and structure of biologically significant glycans	121
	41	Glycosylated naphthalimide	Turn-on	Valuable tool for detecting hexosaminidase activity within cancer cells	122
	42	HMRef αMan	Turn-on	Useful dual-target optical differentiation between malignant and benign tumor	124
	44	β-d-Galactopyranoside linked to the hydroxy group of the naphthalimide	Turn-on	Able to detect β-galactosidase overexpressed cell lines and transient expressed cell lines	125

**Fig. 6:**
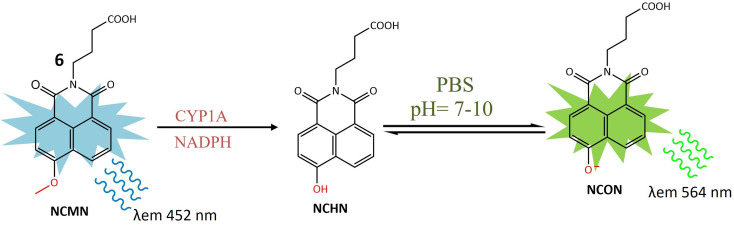
Fluorescence reaction of 6 by CYP1A. NCMN-*O*-demethylation showed a significant fluorescence emission at 564 nm.

A ratiometric two-photon fluorescent probe 6 was designed for the imaging of human CYP1A, responsible for converting procarcinogens into their active carcinogenic forms. The probe, *N*-substituted MN derivative (NCMN) 6, utilized a combination of two-photon microscopy along with ratiometric fluorescence. Change in absorption and emission spectra of 1,8-naphtalimide fluorophore moiety occurred due to dealkylation after interacting with CYP1A (Fig. 6). This results in more tissue specificity. The cleavage and subsequent removal of the methyl group (–*O* demethylation) by CYP1A in the presence of NADPH released *N*-(3-carboxy propyl)-4-hydroxy-1,8-naphthalimide (NCHN), enhancing the red-shift and providing a mechanistic approach for determining the catalytic activity of the specific isoform. After cleavage of the methoxy group of 6 by CYP1A, NCON showed significant fluorescence at emission wavelength 564 nm was observed. The identified metabolite, NCHN, was confirmed through comparisons of LC retention times, UV, and MS spectra with standard samples. The process of *O*-demethylation resulted in a significant increase in fluorescence at 564 nm for the metabolite, accompanied by a gradual decrease in the emission peak at 452 nm for NCMN. Simultaneously, the color of the incubated samples changed from colorless to yellow, indicating that NCMN could serve as a visual colorimetric indicator for the target enzyme(s). Notably, the substantial emission shift towards the red spectrum suggested that this probe could function as a ratiometric fluorescence probe, offering a means to measure the catalytic activity of target enzyme(s) in human biological samples. The catalytic activity of 6 toward both isoforms (CYP1A1 and CYP1A2) was 15 times more than any other human cytochrome enzyme. NCMN has the potential to undergo metabolism, yielding a single metabolite, when exposed to human liver microsomes (HLM) or CYP1A in the presence of a NADPH-generating system under standard physiological conditions (pH 7.4 at 37 °C). To assess its suitability as a CYP1A probe, the performance of NCMN and NCHN was evaluated in phosphate buffer saline (PBS) with varying pH levels from 2 to 10. The fluorescence intensity of both NCMN and NCHN remained largely unaffected by the pH of the medium within the range of 7.0–10.0. This observation suggests that NCMN can effectively operate under physiological conditions. Real-time imaging of human CYP1A was demonstrated in HepG2 and A549 cells, utilizing a methyl ester derivative that could be hydrolyzed by esterases, releasing 6 in the human body. Both 6 and its ester form showed relatively low toxicity in HepG2 and A549 cells. The ester derivative of 6 was also employed for the quantitative analysis of CYP1A in both cell homogenate and intact cells. These findings provided a useful approach for real-time imaging and quantification of CYP1A in biological systems.^44^

### 3.2 Chemosensors responsive to monoamine oxidases

Monoamine oxidase (MAO) is a pivotal enzyme within the human body responsible for the breakdown of various neurotransmitters, including serotonin, dopamine, and norepinephrine. These neurotransmitters play essential roles in the regulation of mood, emotions, and overall mental well-being.^45^ MAO functions by catalyzing the oxidation of monoamine neurotransmitters, converting them into inactive forms that can be safely eliminated from the body. This enzymatic activity is crucial for maintaining the balance of neurotransmitters in the brain, preventing the accumulation of excessive levels that may lead to imbalances associated with various mental health disorders.^46,47^ Additionally, MAO contributes to the metabolism of xenobiotics, which are foreign substances or chemicals entering the body through various means, such as drugs, environmental toxins, and dietary components.^48^ The role of MAO in xenobiotic metabolism varies depending on the specific compound and its chemical properties. While primarily known for its neurotransmitter breakdown in the brain, MAO is also found in other tissues, including the liver and intestines, contributing to xenobiotic metabolism.^49^

**Fig. 7:**
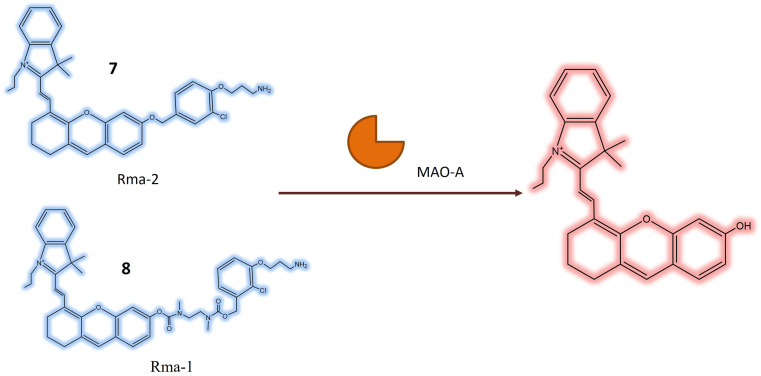
It shows that probes 7 and 8 exhibit increase in fluorescence in the presence of MAO-A.

In a study by Shang *et al.*, two novel near-infrared (NIR) fluorescent probes 7 and 8, were presented for the detection of MAO-A in living biosystems. These water-soluble probes demonstrated selective and sensitive NIR responses specifically towards MAO-A, as opposed to MAO-B. The core structure of the probes included the 3-(2-chlorophenoxy) propan-1-amine unit (Table 1), designed for interaction with MAO-A receptors. To enhance cellular uptake and reactivity with the propylamine unit, a self-immolated linker, a flexible chain that degrades upon entering the cell, was introduced. The ester bond in 7, being more electronegative than the ether bond in 8, influenced the electronic push–pull effect, potentially reducing the background fluorescence of 7 and enhancing its overall functionality. In the absence of MAO-A, probes 7 and 8 initially absorbed light at around 590 and 602 nanometers, respectively. Upon reacting with MAO-A, the maximum absorption peaks of the two probes shift towards approximately 680 nm. This shift is coupled with a 40-fold and 24-fold increase in fluorescence at 708 nm for each probe (Fig. 7), respectively. This suggested that 7 exhibited greater sensitivity to MAO-A compared to 8. Probe 7 was effective in visualizing MAO-A activity within cells, zebrafish, and mice with tumors, indicating its potential for developing new diagnostic imaging tests for carcinoma. Zebrafish treated with 7 exhibited robust fluorescence, and in mice, pre-treatment with clorgyline, an MAO inhibitor, led to a substantial reduction in fluorescence within the tumor area. This provided compelling evidence that the fluorescence observed in the tumor region resulted from MAO-A activity.^50^

**Fig. 8:**
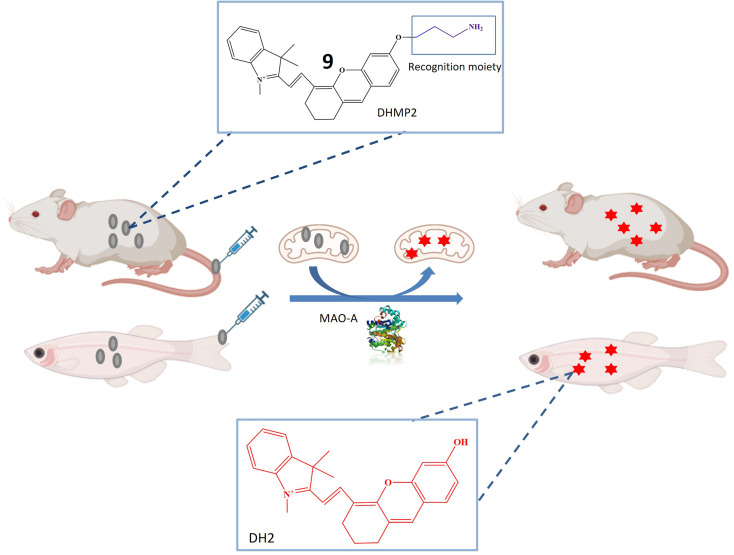
Schematic representation of probe 9 designed for MAO-A detection within cells, zebrafish, and mice with tumors. 9 was mitochondria targeted NIR fluorescent probe that was converted to DH_2_ (increase in fluorescence) after removal of propylamine group.

The research also introduced a novel probe 9, designed for MAO-A detection within cells, zebrafish, and mice with tumors, as illustrated in Fig. 8. This probe exhibited high specificity for MAO-A, making it a valuable tool for quantifying MAO-A activity in intricate biological contexts. The mitochondrial-targeted and near-infrared fluorescent probe could be used to image mitochondria and measure MAO-A activity in living tissues. The mechanism involved selective deprotection of a propylamine group in the presence of MAO-A, leading to the generation of a fluorescent signal. Probe 9 represented a promising advancement in diagnosing and researching diseases linked to MAO-A activity, including cancer, depression, and Parkinson's disease, offering opportunities to explore MAO-A's involvement in various biological processes.^51^

Another innovative fluorescent probe 10, was developed for the specific detection of MAO within cellular environments. This probe showed substantial promise as a valuable tool for investigating MAO's role in neurodegenerative diseases and could potentially pave the way for novel diagnostic and therapeutic approaches for these conditions. The triarylboron core of the fluorescent probe performed its function by undergoing aggregation in the presence of MAO. This aggregation was instigated by the oxidation of a hydrophilic cyclen group located on the probe due to the action of MAO. The resulting shift towards the blue end of its fluorescence spectrum made the probe's fluorescence more pronounced, enabling a ratiometric readout method for assessing MAO activity. This approach offered fast detection of MAOs due to the probe's rapid aggregation process intentionally designed to occur in the presence of MAO. The fluorescence spectra of TAB-2 not only exhibited a noteworthy increase in fluorescence intensity but also experienced a substantial blue shift from 525 nm to 490 nm upon interaction with MAOs.^52^

**Fig. 9:**
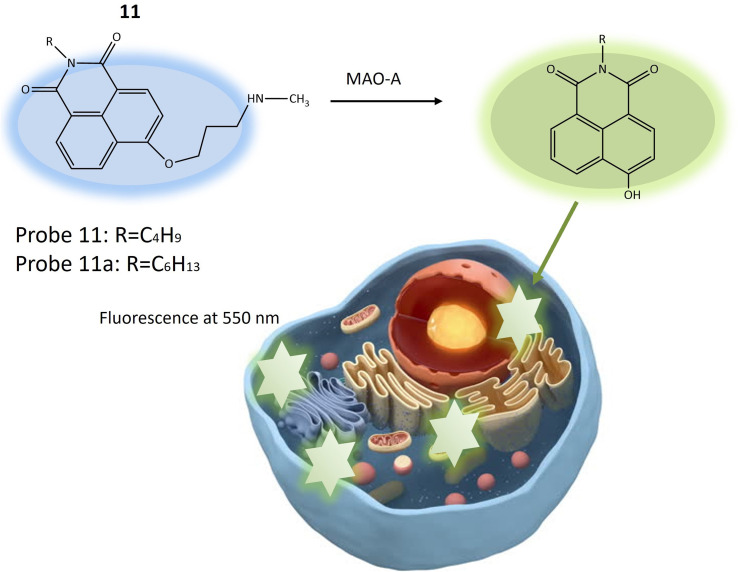
The fluorescence response of 11 and 11a for the monitoring of MAO-A in HeLa cells. Probe 11 and 11a exhibited blue fluorescence and the reaction initiated by MAO-A results in the emission of green fluorescence by releasing 4-hydroxy-1,8-naphthal.

Another fluorescent probe 11a was designed based on the structure of probe 11, with significant potential for investigating neurological conditions like depression and Parkinson's disease, attributed to MAO-A's role in neurotransmitter metabolism and its known association with these disorders. Probe 11a might find utility in exploring various other biological phenomena, including inflammation and cancer, where MAO-A plays a part. The probe was essentially identical to the previous one (11), with a single structural variation related to the length of the alkyl group attached to the nitrogen atom of the 1,8-naphthalimide fluorophore (Table 1). Probe 11 and 11a exhibited blue fluorescence and the reaction initiated by MAO-A results in the emission of green fluorescence by releasing 4-hydroxy-1,8-naphthalimide (Fig. 9). This process leads to a ratiometric fluorescence signal response specific to the enzyme. Experimental findings indicated that probe 11a demonstrates higher reactivity than probe 11 and is notably less susceptible to temperature variations during MAO-A detection. *In vitro* experiments assessed the toxicity of probe 11a using HeLa cells, indicating robust stability with minimal impact on fluorescence signal from changes in pH and temperature. *In vivo* imaging experiments demonstrated the utility of probe 11a for visualizing MAO-A within live zebrafish, offering enhanced precision and reliability compared to conventional single-wavelength imaging. This ratiometric method provided insights into the positioning and dispersion of the fluorescent probe within the specimen, making it a promising tool for biomedical research.^53^

**Fig. 10:**
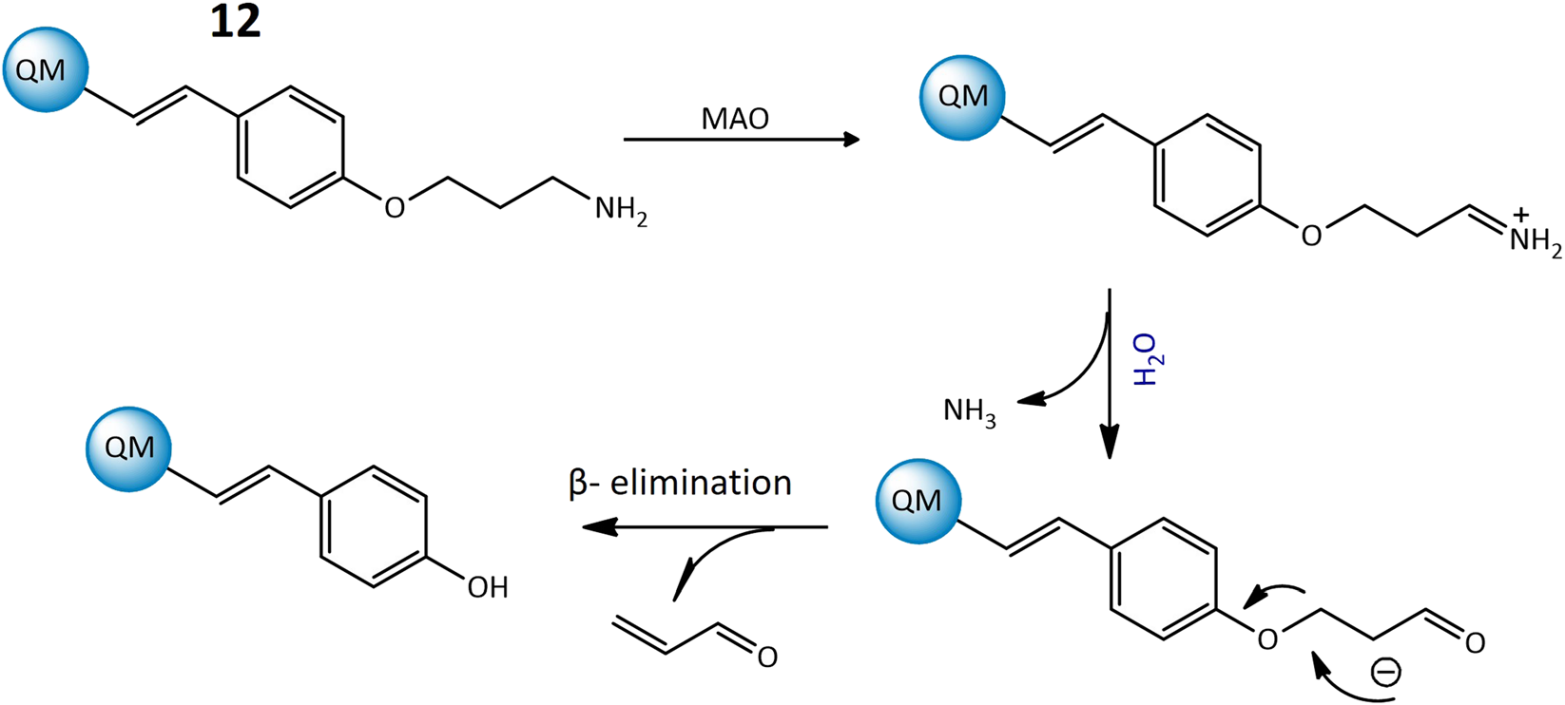
The proposed mechanism of 12 to generate QM-OH from QM-NH_2_ consists of a sequence of steps. Initially, MAOs oxidize the amine within QM-NH_2_, resulting in the formation of an imine. Following this, the imine undergoes a hydrolysis process and aldehyde is formed. The aldehyde participates in a β-elimination reaction, ultimately leading to the formation of QM-OH.

Furthermore, a highly responsive and specific quinoline-malononitrile (QM) based fluorogenic probe, 12, was developed for detecting MAOs in living cells. Probe 12 utilized a β-elimination mechanism upon binding to MAOs, releasing QM-OH (Fig. 10), an aggregation-induced emission that triggered the fluorescence signal, indicating a strong fluorescence emission at 559 nm after interaction with the targeted enzyme. The probe demonstrated rapid response times, providing results within just 5 minutes in both solution and living cells, showcasing its effectiveness for the detection of MAOs in different cancer cells.^54^

### 3.3 Chemosensors responsive to nitroreductase

Nitroreductase is an intriguing enzyme ubiquitously present in diverse living organisms, encompassing bacteria and humans. This noteworthy protein assumes a pivotal role in the metabolism and detoxification of compounds harboring nitro groups.^55^ The principal function of nitroreductase revolves around catalyzing the reduction of nitro compounds, exemplified by nitroaromatics or nitro heterocyclics, through the transfer of electrons. This reduction process transmutes nitro groups into amino groups, thereby converting potentially toxic or harmful molecules into less deleterious forms.^56^ The primary objective of nitroreductase lies in facilitating the biotransformation of xenobiotics featuring nitro groups (–NO_2_). The enzymatic reaction catalyzed by nitroreductase typically entails the reduction of the nitro group to an amino group (–NH_2_), predominantly transpiring under anaerobic conditions.^57^ The intricate mechanism of nitroreductase involves the transfer of electrons to the nitro group, culminating in the reduction of the compound. This electron transfer process is frequently mediated by indispensable cofactors, such as flavin mononucleotide (FMN) or flavin adenine dinucleotide, pivotal for the enzyme's activity. In essence, nitroreductase assumes a critical role in the biotransformation of xenobiotics, facilitating the organism's elimination of potentially hazardous chemicals and contributing substantively to the detoxification processes within the body.^58,59^

**Fig. 11:**
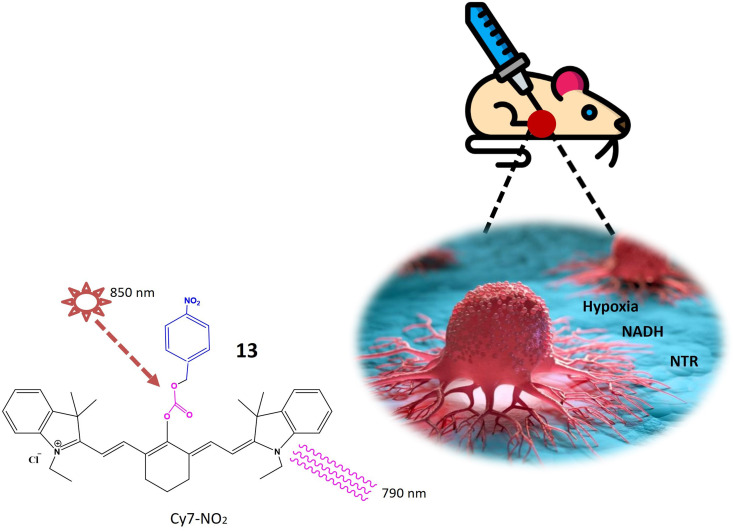
Schematic representation of probe 13 that is specific to NTR and NADH. When excited at 850 nm shown by star, it emits light at a wavelength of 790 nm indicated by curved lines.

Li *et al.* have recently pioneered the development of a cyanine-based fluorescent chemosensor designed for the specific detection of elevated nitroreductase (NTR) levels, leveraging its prevalence as a marker in hypoxic tumors. The chemosensor, denoted as probe 13, exhibits NIR excitation and emission capabilities, enhancing tissue penetration and establishing itself as an exceptional tool for imaging and detection applications. The cyanine structure incorporated into probe 13 imparts near-infrared luminosity. The NTR detection mechanism of probe 13 involves an aromatic nitro group, and the constituent components are intricately linked through an esterification reaction. Upon excitation at 850 nm, probe 13 emits light at 790 nm, as illustrated in Fig. 11. In rigorous *in vitro* experiments, the solution undergoes a rapid color change from green to red, concomitant with a decrease in absorption upon the addition of NTR at a concentration of 0.25 g mL^−1^. The fluorescence intensity at 793 nm notably decreases and shifts to 789 nm through a mechanism referred to as frequency upconversion luminescence (FUCL). In this process, the Cy7-NO_2_ probe, when stimulated at 850 nm, experiences a transition from its excited state to the ground state, resulting in the emission of light at a shorter wavelength, specifically at 789 nm. This occurrence stems from the distinctive electronic transitions occurring within the probe molecule, causing the emitted light to differ from the wavelength of excitation. This unique property facilitates the *in vivo* detection and imaging of NTR, as the emitted light serves as a marker for visualizing the presence of NTR in biological specimens. Functioning as a turn-off probe, probe 13 exhibits a significant alteration in fluorescence intensity within a brief 30 minutes timeframe. Beyond its *in vitro* applications, probe 13 has demonstrated its efficacy in *in vivo* tumor detection. When administered to a mouse model bearing A549 tumors, a remarkable 3.5-fold reduction in fluorescence intensity was observed. This cyanine-based chemosensor proves particularly valuable for detecting hypoxic conditions within solid tumors.^60^

**Fig. 12:**
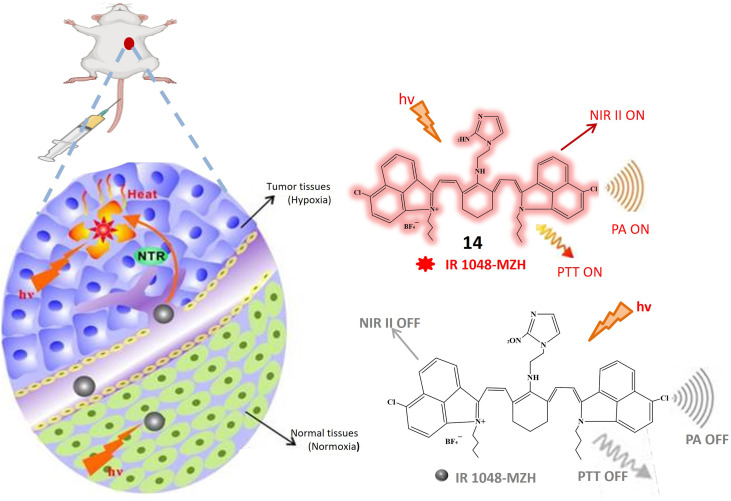
Schematic representation of NIR II fluorescence/photoacoustic probe 14 that responds to NTR and shows increased fluorescence and initiate PTT and PA imaging response through NTR activation.

A recent introduction to the field is fluorescent probe 14, where the imidazole structure serves as an indicator for hypoxic conditions, coupled with IR-1048 utilized as a fluorescence detector. In rigorous *in vitro* investigations, probe 14 manifests itself as a turn-on probe. Upon the addition of the NTR solution, discernible enhancements in absorption and emission wavelengths at 980 nm and 1046 nm, respectively, were observed. The emission wavelength demonstrated a remarkable increase of nearly 106.9 times. A notable advantage of probe 14 lies in its absence of signal-to-noise ratio issues in *in vivo* studies. In tumor cells, probe 14 increases its fluorescence and PTT and PA imaging is on as depicted in Fig. 12. Photothermal studies of hypoxic tumor regions exhibited green fluorescence, indicating an elevated level of NTR. Additionally, this study incorporated 3D photoacoustic imaging, contributing to the determination of tumor tissue penetration depth. The 3D photoacoustic signals revealed a longitudinal depth of the tumor tissue at 14.6 ± 0.2 mm, 14 hours post-injection of the probe. Photoacoustic Tomography is an imaging technique that combines the principles of ultrasound and laser-induced photoacoustic effects. While Photothermal Therapy involves using laser-induced heat to selectively destroy or damage target tissues, typically for therapeutic purposes. Photoacoustic Tomography is a powerful imaging technique, while Photothermal Therapy is a therapeutic approach. This integration allows for precise targeting, monitoring treatment progress, and assessing therapeutic outcomes.^61^

**Fig. 13:**
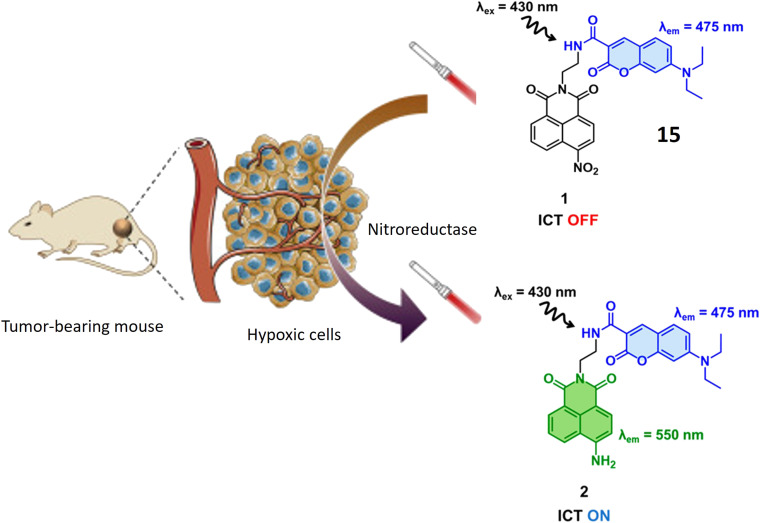
Schematic representation of probe 15 that displays enhanced fluorescence specifically in hypoxic cells present in tumors. Within this probe, the coumarin part serves as an internal reference, emitting light at 475 nm, while the nitro-naphthalimide section shows increased fluorescence at 550 nm when the nitro probe exhibits an elevation in fluorescence, and this response is attributed to its sensitivity to two unique features found in cancer cells: low pH levels and the presence of NTR.

Yoon *et al.* have introduced a bifurcated fluorescent chemosensor denoted as probe 15, proficient in emitting fluorescence from both of its constituent components. The probe comprises two integral parts: coumarin, inherently possessing fluorescence, and nitro-naphthylamide, which becomes fluorescent upon activation by the enzyme NTR. Notably, NTR assumes a pivotal role in the conversion of nitro groups into amino groups, exhibiting notably elevated concentrations in hypoxic cells, where it actively participates in the metabolism of diverse xenobiotics. Functioning as a turn-on probe, 15 experiences a substantial increase in fluorescence activity in the presence of NTR, rendering it particularly effective for the detection of NTR activity in cancerous and hypoxic tissues, as illustrated in Fig. 13. In the presence of NTR, coumarin fluorescence is observed at 475 nm, while the nitro-naphthylamide component emits fluorescence at 550 nm in the presence of NADH. These specific wavelengths facilitate the precise detection of NTR activity and enable the assessment of metabolic processes. Probe 15 exhibits biocompatibility with both normal NIH 3T3 cells and cancerous HeLa cells, ensuring its utility for fluorescence monitoring and detection in both cancerous and normal live cells without inducing toxic effects. ICT is a process where an excited electron migrates from one part of a molecule to another. In the presence of NTR, there is relocation of an electron within 15 (nitro group changes to amino group), leading to charge separation. This phenomenon is known as ICT. Fluorescent probes based on Intramolecular Charge Transfer mechanisms are valuable tools for sensing and imaging applications.^62^

**Fig. 14:**
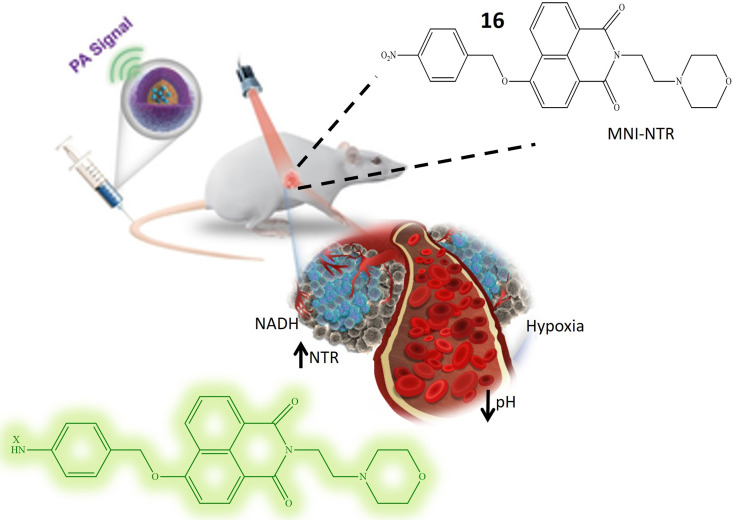
It illustrates that the probe 16 exhibits an elevation in fluorescence, and this response is attributed to its sensitivity towards low pH level and presence of NTR.

Li *et al.* and their research team have developed a highly versatile probe 16 designed to detect both pH levels and NTR activity. This adaptable probe is centered around a naphthylamide core and incorporates specific functional groups for detecting pH and NTR. In a neutral solution with a pH around 7, the probe remains non-fluorescent. However, as the pH decreases, the probe undergoes protonation at the nitrogen atom in the attached morpholine group, resulting in the emission of blue fluorescence. The probe exhibits weak green fluorescence in hypoxic tissues characterized by low oxygen levels and typically high NTR levels. Importantly, in environments featuring both low pH and elevated NTR levels, the probe emits strong green fluorescence. This dual response highlights its capability to detect acidic conditions and raised NTR concentrations, as depicted in Fig. 14. Upon the addition of NTR and NADH, a fluorescence peak at 524 nm is observed, originating from the reduction of the nitro benzyl group by NTR. In acidic environments, the fluorescence intensity at 524 nm increases, indicating sensitivity to both NTR concentration and pH. The absorption and emission wavelengths increase with higher concentrations of NTR enzymes and lower pH values, demonstrating a proportional response of fluorescence to NTR concentration and environmental acidity. Considering that tumor cells frequently exhibit elevated NTR concentrations and an extracellular acidic environment, distinctive characteristics of cancer cells, this probe holds significant practical value in biological applications. Particularly, it can be utilized in tumor fluorescence imaging to detect acidity levels and evaluate NTR expression in live cells.^63^

**Fig. 15:**
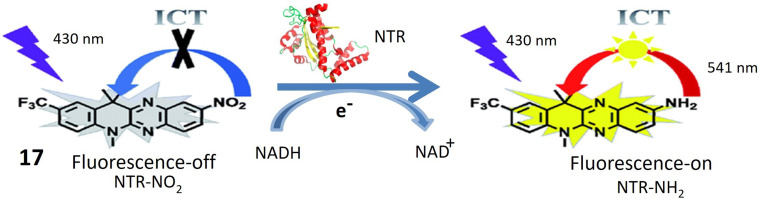
Demonstrates that probe 17 emits fluorescence when exposed to NTR and NADH in an *in vitro* setting.

Probe 17, designed by Jia *et al.* emerges as a promising tool for NTR detection, holding potential applications in diagnosing or detecting tumor cells in live cellular environments. NTR-NO_2_ features a quinoxaline ring within its structure, a structural attribute anticipated to play a pivotal role in facilitating interaction with NTR and inducing fluorescence changes upon exposure to NTR and NADH. Notably, probe 17 exhibits a substantial 30-fold increase in fluorescence when subjected to a combination of NTR and NADH. This pronounced enhancement in fluorescence signals the utility of 17 as a fluorescent probe for detecting the presence of NTR. The conversion of the nitro group in 17 into an amino group stands out as a key reaction triggered by the presence of NTR. This reaction likely underlies the observed increase in fluorescence. Described as biocompatible, probe 17 can be safely employed in living organisms without causing harm or interference with biological processes. Its photostability denotes the capability to maintain fluorescence properties over an extended period and under light exposure. Probe 17 exhibits high selectivity, as evidenced in Fig. 15, confirmed by its reactions with NaBH_4_ and NADH. This selectivity underscores the probe's specific targeting of NTR and its responsiveness to its presence, thereby minimizing the likelihood of false-positive results. The experiment conducted on HeLa cells demonstrates that 17 can effectively detect tumor cells. The probe likely responds to the presence of NTR in these cells, positioning it as a potential tool for cancer diagnosis or tumor cell detection. However, further research and validation may be necessary before widespread adoption in clinical or research applications.^64^

### 3.4 Chemosensors responsive to aldehyde dehydrogenases

Aldehyde dehydrogenases (ALDHs) represent a critical class of NADP^+^-dependent enzymes integral to the conversion of both endogenous substrates (*e.g.*, amino acids and lipids) and exogenous compounds (*e.g.*, xenobiotics and drugs) from aldehydes to their corresponding carboxylic acids.^65,66^ Comprising 11 families, this enzyme superfamily plays a vital role in various physiological processes, and deviations in its levels are linked to numerous life-threatening diseases.^67^ Notably, reduced ALDH levels are associated with neurodegenerative conditions, while increased levels are often observed in cancer cells. Elevated ALDH levels, particularly in ovarian, lung, liver, and breast cancer cells, are potential indicators of malignant tumors.^68^ Moreover, high ALDH levels contribute to increased resistance of cancer cells to cytotoxic drugs and other treatment modalities, exemplified by the resistance conferred by overexpression of ALDH1A1 and ALDH3A1 to cyclophosphamide.^69,70^ The ALDH1 isoenzyme serves as an identification marker in both normal and cancer stem cells, underscoring the importance of monitoring ALDH activity in cancer cells for targeted therapeutic interventions.^71^

**Fig. 16:**
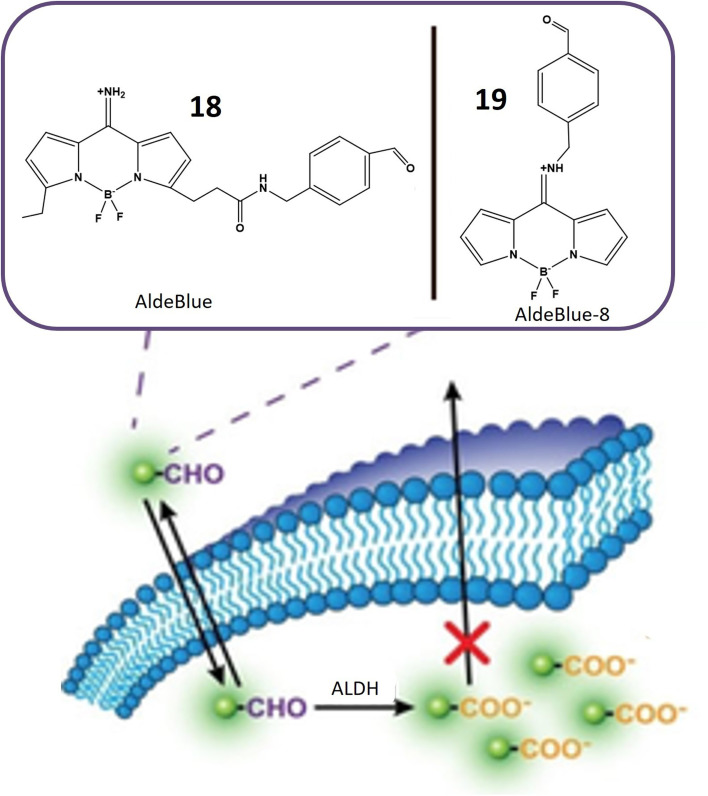
Schematic representation of probes 18 and 19 that can easily move through cell membranes and get trapped inside cells. ALDH enzyme oxidizes the aldehyde part of the probe, changing it into a carboxylate ion. This transformation causes the probe to become confined within the cell, where it shows increased fluorescence.

Probes 18 and 19, developed by Yagishita and his colleagues, represent a significant leap in the realm of ALDH detection. These probes distinguish themselves as the first probes capable of traversing cell membranes freely and becoming entrapped within cells exhibiting ALDH activity, as depicted in Fig. 16. This distinctive property positions them as valuable tools for *in vivo* imaging of ALDH activity. Notably, these probes exhibit exceptional sensitivity and specificity for ALDH and find application across diverse cell types and tissues. Anchored on a BODIPY fluorophore with an amino methyl benzaldehyde moiety (Table 1), these probes derive their fluorescence from the BODIPY fluorophore, while the amino methyl benzaldehyde moiety imparts specificity for ALDH. ALDH catalyzes the oxidation of the aldehyde segment of the probe, converting it into a carboxylate ion and leading to its entrapment within the cell, rendering it detectable. The probes are available in various colors, including green, red, and NIR, catering to a range of applications. The excitation and emission wavelengths of these probes may vary based on environmental factors, and they are typically utilized with fluorescence microscopes or flow cytometers. A notable advantage of these probes is their compatibility with commercially available red and green probes, facilitating the detection of ALDH in the presence of background fluorescence.^72^

**Fig. 17:**
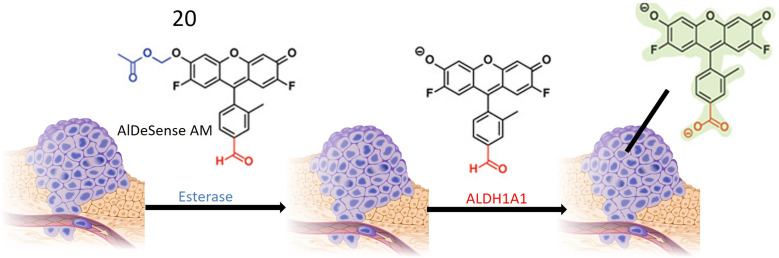
Schematic representation of probe 20 that is initially converted into AlDeSense by an esterase enzyme. Eventually, a fluorescent metabolite is produced through the action of A1DH1A1, which is present at elevated levels in tumor cells.

Chemosensor 20, devised by Anorma *et al.* and their research team, represents a noteworthy contribution to the realm of ALDH1A1 sensing. This chemosensor incorporates Pennsylvania Green dye coupled with a benzaldehyde moiety, specifically engineered for the detection of ALDH1A1. Functioning as a turn-on probe, 20 exhibits an increase in fluorescence intensity upon interaction with its target, ALDH1A1, as illustrated in Fig. 17. This unique property enables the sensitive detection of ALDH1A1 activity. Demonstrating high selectivity, chemosensor 20 exhibits minimal cross-reactivity with other ALDH isoforms, a crucial characteristic for precise detection and monitoring of ALDH1A1 activity in biological specimens. The incorporation of a methyl acetate group enhances the chemosensor's cell permeability, facilitating its entry into cells where interaction with ALDH1A1 occurs. Upon oxidation of the aldehyde moiety within 20 to a carboxylic acid, the probe emits fluorescence at a wavelength of 516 nm, providing a clear indication of ALDH1A1 activity within the cells. Extensive testing on various cancer cell types, including K562 (a leukemia cell line), mammospheres, and B16F0 melanoma cells, has revealed elevated ALDH1A1 levels in cancer stem cells (CSCs). The probe exhibits robust fluorescence when exposed to these cells. To confirm the specificity of 20, fluorescence comparisons were made between cancer cells with siALDH1A1 knockdown and ALDH1A1-lacking HEK293T cells, further affirming the probe's selectivity for ALDH1A1. Notably, the chemosensor has been successfully employed for *in vivo* imaging in live cells, showcasing its potential in detecting ALDH1A1-enriched CSCs within living organisms. This application holds significant promise for advancing cancer diagnosis and treatment strategies.^73^

**Fig. 18:**
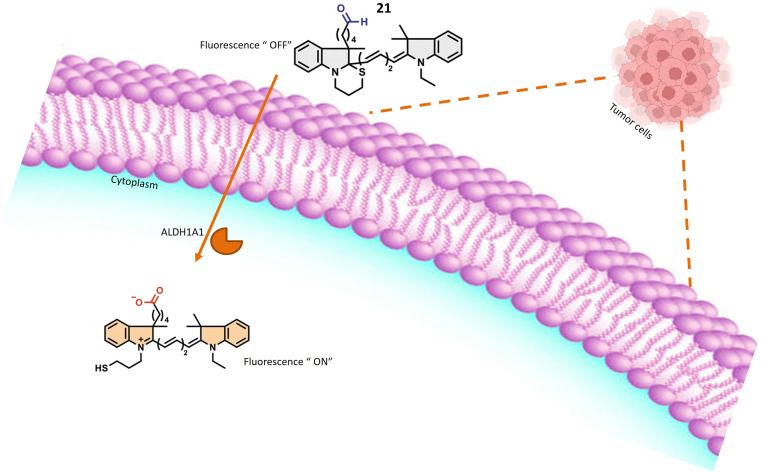
It indicates that in the presence of A1DH1A1, the probe exhibits fluorescence within tumor cells.

Probes 21 and 22, developed by Oe *et al.* and their collaborators, were meticulously crafted with a benzoindole-based fundamental structure (Table 1). These probes were strategically engineered for the specific detection of ALDH1A1 activity, particularly within various cancer cell lines, as elucidated in Fig. 18. The probes exhibit distinct excitation wavelengths that undergo shifts upon interaction with ALDH1A1. In their unbound states, 21 and 22 display excitation wavelengths of 650 nm and 740 nm, respectively. However, in the presence of ALDH1A1, the emission wavelengths shift to 665 nm for 21 and 770 nm for 22 and fluorescence intensity at these wavelengths increases significantly. This alteration in fluorescence characteristics is attributed to the dynamic interaction between the probes and ALDH1A1, resulting in a discernible and quantifiable signal. *In vitro* assessments were conducted utilizing different cancer cell lines, including SUIT-2, A549, and H1299, representing pancreas, lung, and gastric adenosquamous cancers, respectively. These experiments aimed to evaluate the presence and activity of ALDH1A1 in these specific cancer cell lines. The probes' capacity to undergo changes in excitation wavelength and exhibit an increase in fluorescence intensity upon interaction with ALDH1A1 positions them as valuable tools for probing the role of ALDH1A1 in diverse cancers. Furthermore, their potential application in diagnostic and research endeavors within the field of oncology is noteworthy. ALDH1A1, a member of the aldehyde dehydrogenase (ALDH) family, is recognized for its role in the oxidation of aldehydes to form their respective carboxylic acids. Furthermore, ALDH1A1 is implicated in the ring structure opening of probes, leading to a notable enhancement in their fluorescence intensity. The precise mechanism involves the enzymatic binding of the substrate (the fluorescence probe) to the active site of ALDH1A1, followed by the catalytic oxidation of the aldehyde group to form a carboxylic acid. This likely entails the hydride transfer from the aldehyde to the cofactor NAD+ (nicotinamide adenine dinucleotide), resulting in the generation of NADH and the corresponding carboxylic acid product. Elucidating the intricate details of how ALDH1A1 initiates the ring structure opening of the probes and catalyzes the aldehyde-to-acid conversion involves comprehensive investigations such as enzymatic kinetics studies and structural analyses. Probes 21 and 22 work on AiQd (activator-induced quencher-detachment) mechanism. The AiQd mechanism is a fluorescence turn-on strategy that involves the use of a quencher molecule that is attached to a fluorophore through a specific chemical bond. In the absence of the target analyte, the quencher molecule effectively suppresses the fluorescence of the fluorophore, resulting in a low background signal. However, in the presence of the target analyte, the quencher molecule is detached from the fluorophore, leading to a significant increase in fluorescence intensity and a turn-on response.^74^

**Fig. 19:**
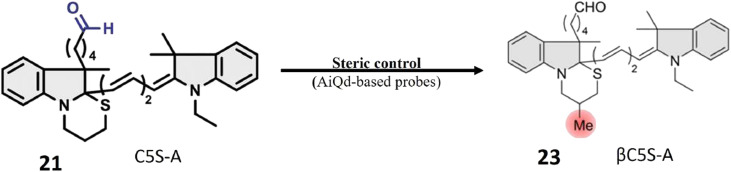
It demonstrates the creation of 23 from 21 through a process involving steric control and the AiQd mechanism. To achieve steric control, a methyl group was added to the β-position of the –SH group. This modification brings several advantages over the previous probe, such as a significant increase in the signal-to-noise ratio (>10-fold) and a decrease in the response to biological thiols. These enhancements are vital for enhancing the sensitivity and precision of ALDH1A1 detection and providing a high-contrast visualization of cancer stem cells.

Oe *et al.*'s development of the novel turn-on ALDH1A1-responsive probe 23 signifies a significant advancement over its predecessors, addressing challenges related to false background signals and signal-to-noise ratio. Probe 23 operates on AiQd mechanism, wherein a carboxylic group serves as the activator, and a thiol (–SH) group functions as the nucleophilic quencher, with an additional methyl group strategically positioned at the β-position of the –SH group for steric control, as illustrated in Fig. 19. The presence of the methyl group is pivotal to the probe's functionality. In the absence of ALDH1A1, the methyl group facilitates the maintenance of the closed-ring structure of the probe in a chair form, preventing steric repulsion induction. However, upon encountering ALDH1A1, the ring structure of the probe opens, and the methyl group induces steric repulsion. This steric repulsion constitutes a crucial element in the probe's response to ALDH1A1 activity. A notable improvement with probe 23 lies in its reduced susceptibility to generating false background signals, achieved by minimizing interactions with biological thiols that could lead to inaccurate fluorescence readings. The steric repulsion induced by the methyl group effectively prevents such interactions, ensuring a more precise assessment of ALDH1A1 activity. The modifications, including the introduction of steric control and the AiQd mechanism, contribute to an enhanced signal-to-noise ratio, thereby increasing the sensitivity and accuracy of ALDH1A1 detection. The probe has been successfully employed for Cancer Stem Cell (CSC) identification in SUIT-2 cells, a pancreatic cancer cell line. The robust fluorescence observed in these cells is attributed to the heightened activity of ALDH1A1, establishing βC5S-A as a valuable tool for identifying and studying CSCs in this specific context. Probe 23 represents an innovative approach to detecting ALDH1A1 activity with improved specificity and reduced background noise, positioning it as a promising tool for the identification and study of cancer stem cells.^75^

**Fig. 20:**
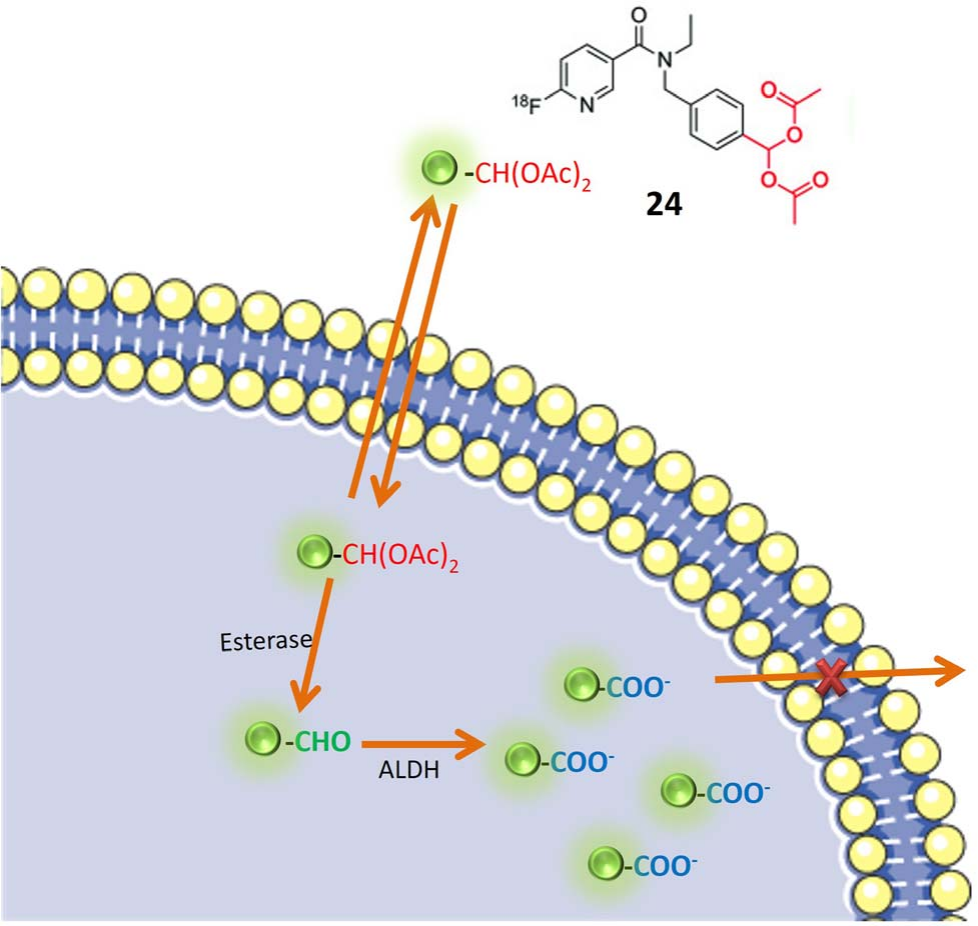
It depicts a specialized probe 24 designed for ALDH1A1, which is a marker linked to drug-resistant cancer cells. This probe is initially dormant but is activated by esterase enzymes within cells. Once activated, ALDH1A1 modifies the probe into a carboxylate ion, causing it to be trapped inside the cell.

Pereira and colleagues devised a radioactive aromatic aldehyde probe, denoted as 24, designed for ALDH1A1 imaging through the implementation of a prodrug strategy. The probe, initially inert, undergoes activation by esterase enzymes within cells. Upon activation, ALDH1A1 facilitates the transformation of the probe into a carboxylate ion, leading to its entrapment within the cellular milieu, as illustrated in Fig. 20. This innovative approach resulted in the development of a specific probe tailored for ALDH1A1, a recognized marker linked to resistant cancer cells. To ensure the probe's specificity for ALDH1A1, Witney *et al.* and colleagues employed an acylal group to shield the aromatic aldehyde from systemic metabolism. This protective mechanism was instrumental in averting nonspecific interactions or metabolism of the probe outside the intended ALDH1A1 pathway. The probe's selectivity for tumor-associated ALDH was evaluated using two ovarian cancer cell lines, SKOV3-ip1 and SKOV3-TRip2. Notably, SKOV3-TRip2 cells, characterized by heightened ALDH1A1 expression, exhibited increased fluorescence in the Aldefluor assay. This observation attests to the effective trapping of the probe in cells with elevated ALDH1A1 expression. Pharmacological inhibition of ALDH activity resulted in reduced radiotracer retention in cancer cells, providing additional confirmation of the probe's specific association with ALDH activity. *In vivo* positron emission tomography (PET) imaging of mice harboring ovarian tumors demonstrated tumor-specific uptake of the radiotracer. However, the imaging modality was unable to differentiate between tumors exhibiting high and low ALDH expression levels. Although Witney and colleagues' radioactive aromatic aldehyde probe exhibited promising outcomes for tumor detection, the ability to distinguish between tumors with varying ALDH expression levels within the same imaging context may necessitate further optimization or the application of complementary imaging techniques.^76^

### 3.5 Chemosensors responsive to esterases

Carboxylesterases (CESs), integral members of the serine hydrolase family, have garnered extensive applications in pharmaceuticals, biotechnology, and environmental sciences.^77^ These enzymes exhibit widespread expression in barrier tissues and organs such as the brain, liver, skin, small intestine, and lungs.^78,79^ Functionally, CESs catalyze the hydrolysis of ester-containing compounds, yielding alcohols and acids.^80^ Their pivotal roles extend to the metabolism of xenobiotics, signal transduction, bioactivation of prodrugs, and the regulation of lipid homeostasis.^81^ Dysregulation of esterase homeostasis is associated with various pathological conditions, including obesity, cancer, neurodegenerative disorders, and Wolman disease.^82^

**Fig. 21:**
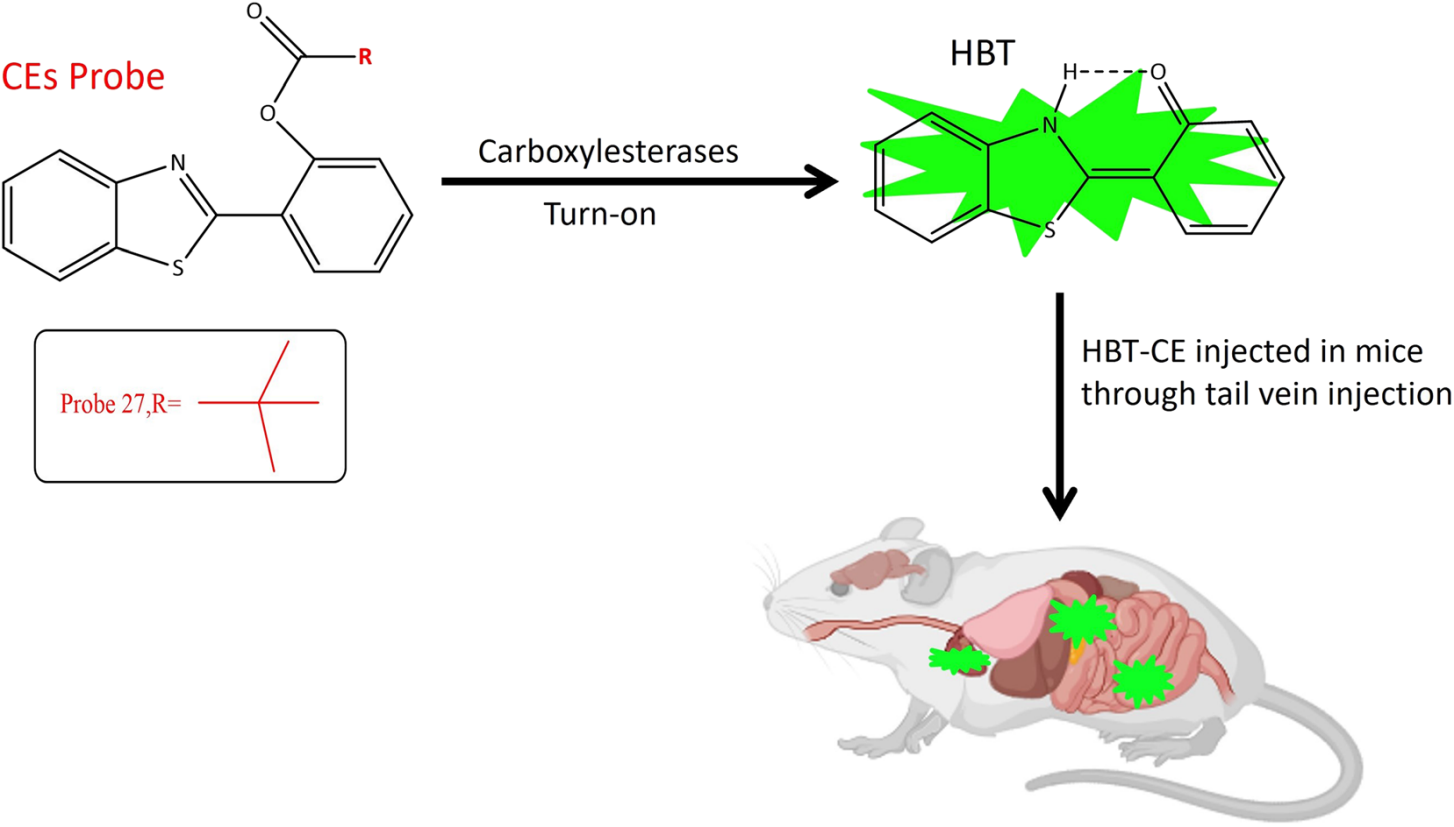
It demonstrates that probe 27 exhibits increase in fluorescence upon interaction with the targeted enzyme and the biodistribution of CES in mice.

Understanding the activation mechanism and function of carboxylesterases is paramount for drug discovery and development.^83^ Over the past decades, numerous fluorescent probes for carboxylase detection have been reported.^84^ A notable tool for the detection and tracking of endogenous CES is probe 27. The fluorophore moiety, 2-(2-hydroxyphenyl)benzothiazole (HBT), exhibits excellent biocompatibility and a substantial Stokes shift (Table 1). Three distinct alkanoyl groups of varying sizes were incorporated as recognition components in the HBT for the design of the target probes: probe 25 featured an ethyl group, probe 26 incorporated an isopropyl group, and probe 27 included a tertiary butyl group. All three probes exhibited similar responses when exposed to both carboxylesterases. Importantly, as the steric hindrance of the recognition group increased, sensitivity to carboxylesterases decreased. However, their specificity in distinguishing from butyrylcholinesterase (BChE) improved, with probe 27 demonstrating the highest level of specificity and fluorescence at 460 nm.^10^ Chemosensor probe 27 targeted both isoforms, CE1 and CE2, with the pivaloyl group serving as the recognition site, providing superior specificity compared to other small molecule probes with an acetyl group as the recognition site (Table 1). The pivaloyl group also imparted steric hindrance to other esterases, including butyrylcholinesterase (BChE) and acetylcholinesterase (AChE). Cellular imaging and kinetic parameters were determined by applying probe 27 to Hep-G2 cell lysates, showcasing an increase in intracellular signals in a time-dependent manner. Biodistribution of carboxylesterases was assessed by injecting probe 27 into nude mice *via* tail vein injection, revealing a significant fluorescence signal in the abdomen. *In situ* imaging of major organs disclosed the distribution of carboxylesterases in the lung, small intestine, and liver *via* fluorescence signals, with no signal observed in the kidney, heart, and spleen, as depicted in Fig. 21. These findings underscore the utility of probe 27 as a robust tool for detecting and monitoring endogenous carboxylesterase imaging and biodistribution in intricate biological systems.^51^

**Fig. 22:**
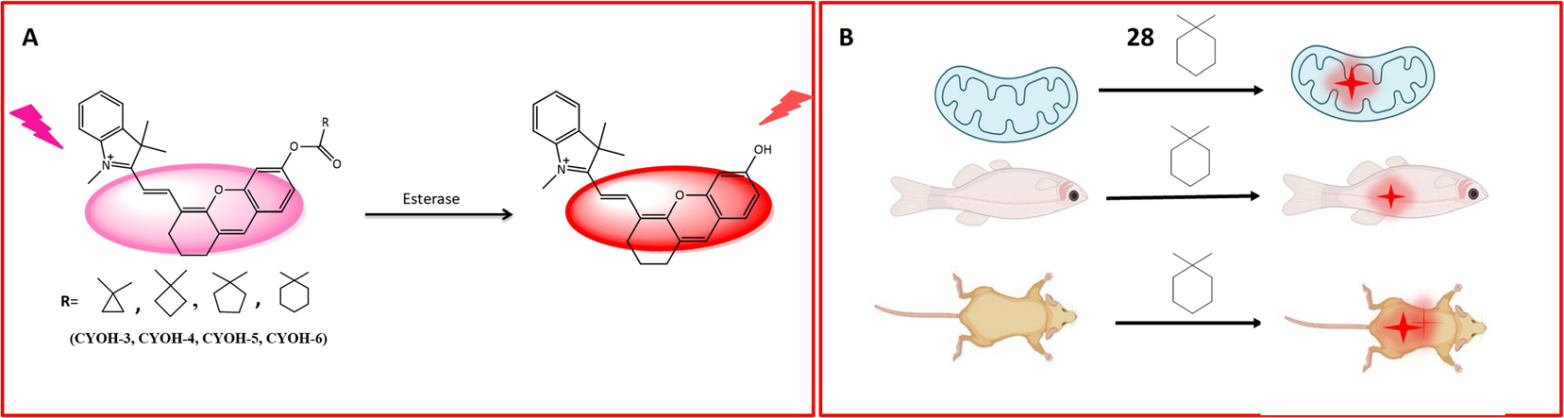
Schematic representation of (A) fluorescence activity of the probe with different R groups towards esterases and (B) 28 to determine the biodistribution of esterase in live cells and zebrafish and determination of the toxicity of 28 in mice that showed no toxicity.

A multifunctional near-infrared fluorescent probe 28 was meticulously designed to enable the visualization of *in vivo* esterase activity within mitochondria. This involved a strategic alteration of the cyclic ring structure of CYOH (fluorophore) to generate a series of near-infrared (NIR) fluorescent probes. The design incorporated indole and dihydroxy xanthene, along with cyclic ring structures such as cyclopropyl, cyclobutyl, cyclopentyl, and cyclohexyl, serving as the recognition elements for carboxylesterase (CE), as delineated in Fig. 22A. By modifying the ring structure of the detection moiety from three carbon to six carbon, corresponding changes in fluorescence emission were observed. The probes, facilitated by ester hydrolysis, underwent changes in emission spectra, revealing the fluorophore moiety and providing ratiometric readouts. To assess the localization of esterase activity in mitochondria, HeLa cells were employed. Among the probes, probe 28 exhibited optimal fluorescence absorption and emission at 585 nm and 561 nm, respectively (Table 1). Systematically altering the triggering group from cyclopropyl to cyclohexyl resulted in corresponding changes in wavelength emission properties (650, 654, 658, and 661). Further investigations involved the evaluation of esterase activity in zebrafish. Confocal imaging, conducted post a 60 minutes incubation period, revealed the biodistribution of esterase in the head, abdomen, and ventral tail of the zebrafish. Probe 28's toxicity was assessed by injecting the probe into live healthy mice, with fluorescence signals recorded at intervals of 0, 5, 10, 30, and 60 minutes. When excited by light at 690 nm, probe 28 exhibited robust fluorescence in the 705–750 nm range for *in vivo* esterase detection. Importantly, this fluorescence remained unaffected by ambient light, indicating the absence of toxicity, as depicted in Fig. 22B. These findings underscore the successful utility of probe 28 for the detection of esterase in mitochondria and real-time imaging.^77^

**Fig. 23:**
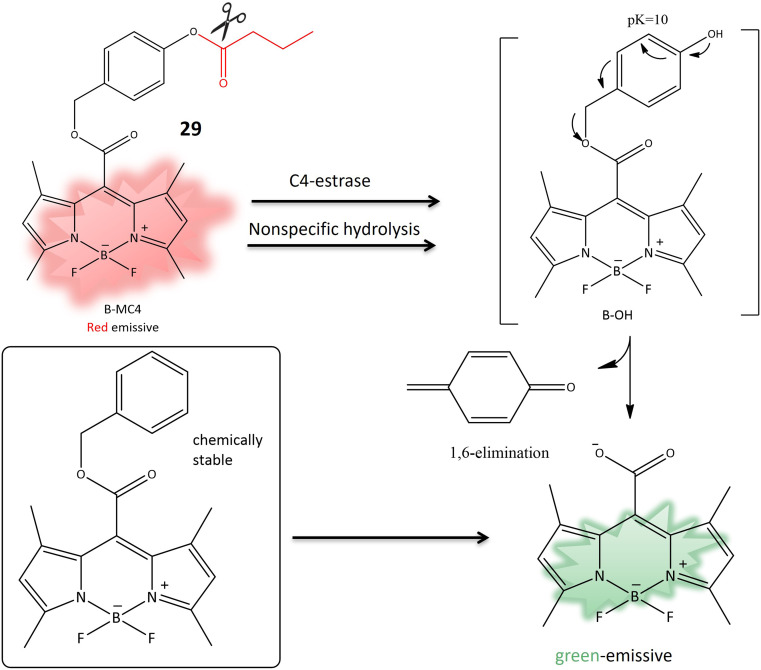
The transformation of 29 to meso-carboxylate BODIPY is initiated by the action of C4-esterase, resulting in a fluorescence change, used to detect the presence of *M. atarrhalis*.

Another coumarin-based fluorogenic probe 29, incorporating a boron dipyrromethene (BODIPY) dye (Table 1), was developed for the identification of bacteria containing butyrate esterase, particularly targeting *Moraxella catarrhalis*, a Gram-negative diplococcus found in the human respiratory tract. The design involved substituting the meso-ester with meso-carboxylate, incorporating a *p*-(butyryloxy) benzyl group (B-MC4) (Table 1). This modification resulted in strong green emission in aqueous media due to the elimination of 1,6-quinone-methide through self-immolation (Fig. 23). The probe utilized phenyl butyrate for butyrate esterase recognition. The enzyme-dependent hydrolysis induced red fluorescence, followed by the release of strong green emission in aqueous media, demonstrating the effectiveness of B-MC4 for the rapid and precise detection of *Moraxella catarrhalis*.^85^

Two probes, 30 and 31, utilizing the dye 6-amino-1-phenalenone in conjunction with benzoyl (1) and pyruval (2), were designed to assess the activity of human carboxylesterase 2 (HCES2) in cultured pancreatic cancer cells. The choice of dye was motivated by the conjugation of the exocyclic amine with the pi-bond of the dye to evaluate the effectiveness of chemotherapy. The absorption spectrum exhibited a blue shift upon conversion of the amine to an amide through ligation to a benzyl or pyruval moiety (Table 1). Hydrolysis of the amide bond by human carboxylesterase 2 induced a red shift in the absorption spectrum. Both probes demonstrated a change in fluorescence from red to yellow shift, indicating the recovery of fluorescence activity. Probe 30, specifically targeting HCES2 activity in pancreatic cancer cells, showcased its effectiveness as a water-soluble, photostable ratiometric chemosensor for detecting HCES2 in the context of pancreatic cancer.^86^

**Fig. 24:**
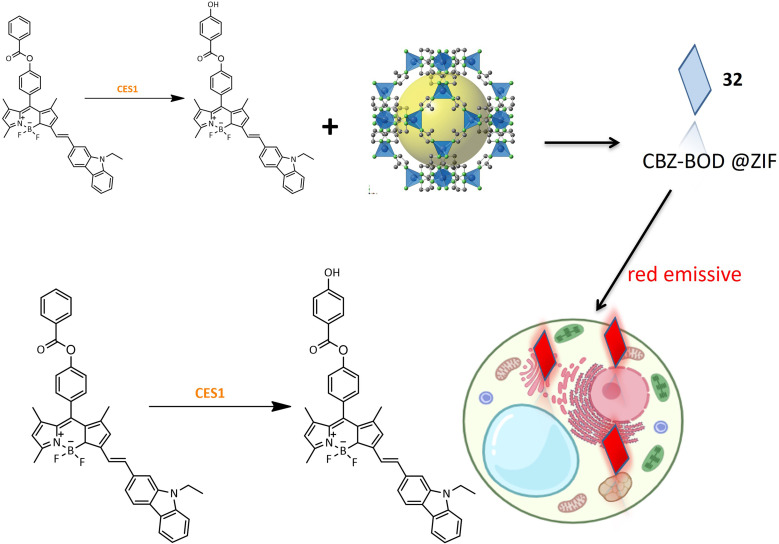
Designing and the fluorescence activity of 32 in HepG2 cells.

A red emission fluorinated boron-dipyrromethene (BODIPY) based probe 32 was designed for the detection of carboxylesterase 1 (CES1) activity in living cells. The small organic molecular probe 32 was encapsulated within a metal–organic skeleton using zeolite imidazolate framework-8 (ZIF-8) to enhance biocompatibility and stability. The synthesis involved conjugating the fluorophore part of BODIPY with *N*-ethyl-3-carbazolecarboxaldehyde as an electron donor, and benzoyl chloride was introduced to BOD-OH through an ester linkage (Table 1). Probe 32 exhibited specificity toward CES1 with a sharp fluorescence shift, demonstrating its potential for bioimaging of CES1 (Fig. 24). Moreover, probe 33, derived from probe 32, exhibited improved detection properties, highlighting the potential for designing new probes with enhanced specificity.^87^

### 3.6 Chemosensors responsive to aminopeptidase

Aminopeptidase is an enzymatic catalyst that plays a pivotal role in the intricate processes of protein digestion and metabolism. Specifically, aminopeptidases are integral to the hydrolysis of peptide bonds situated at the N-terminus (the amino end) of a polypeptide chain. This enzymatic hydrolysis results in the sequential removal of amino acids from the peptide chain.^88,89^ Aminopeptidases exhibit diverse roles in pathological conditions, with relevance to their involvement in tumor development and progression. In the context of cancer, these enzymes are implicated in facilitating cancer cell invasion into surrounding tissues and metastasis to distant body parts. Additionally, aminopeptidases contribute to angiogenesis by cleaving proteins associated with the regulation of blood vessel formation.^90,91^ The expression and activity levels of specific aminopeptidases have been identified as valuable prognostic indicators in certain cancer types. Consequently, aminopeptidases have emerged as potential therapeutic targets for cancer treatment. Strategic inhibition of specific aminopeptidases presents an approach to impede cancer cell proliferation and invasion.^92,93^ In recent years, there has been noteworthy progress in the development of fluorescent chemosensors tailored for the identification and quantification of various enzymes, including amidases. This encompasses a range of aminopeptidases such as aminopeptidase N (APN), alanine aminopeptidase (AAP), γ-glutamyl transpeptidase (GGT), *N*-acetyl-d-glucosamine (NAG), fatty acid amide hydrolase (FAAH), leucine aminopeptidase (LAP), and pyroglutamyl aminopeptidase I (PGP-1).^94^

**Fig. 25:**
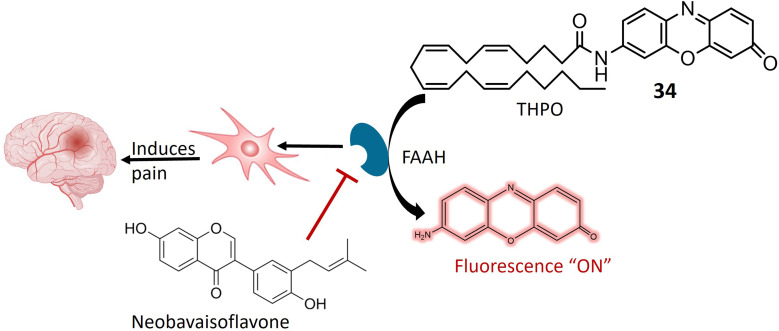
Suggests that FAAH enzyme plays a pivotal role in pain regulation in the body. A fluorescent probe 34 is designed to specifically interact with and be cleaved by FAAH, exhibiting a high degree of sensitivity and remarkable specificity for this enzyme. Additionally, the figure demonstrates the inhibitory effects of neobavaisoflavone on FAAH.

Anandamide, classified as an endocannabinoid, belongs to the group of fatty acid amides and is metabolized by the enzyme fatty acid amide hydrolase (FAAH). Anandamide plays a pivotal role in various physiological processes, including appetite regulation, mood modulation, and pain sensation.^95,96^ Inhibiting FAAH can elevate the production of anandamide and other fatty acid amides, potentially leading to therapeutic advantages. Consequently, FAAH is a crucial target for medication development, especially for conditions involving inflammation, pain, and anxiety.^97^ A specialized fluorescent probe, denoted as 34, was specifically designed to target and be cleaved by FAAH, as illustrated in Fig. 25. Probe 34 offers several advantages over alternative screening methods, such as luciferase-dependent probes. Utilizing fluorescence spectroscopy facilitates the identification and quantification of the fluorescent substance generated when FAAH cleaves 34. Under physiological conditions, the metabolite of 34 exhibits consistent and unchanging fluorescence emission. Notably, the extended excitation and emission wavelengths of 34 enable greater tissue penetration and reduced interference from auto-fluorescence background. Additionally, it demonstrates high sensitivity and exceptional selectivity for FAAH, enabling the detection of FAAH activity in complex biological systems. In *in vitro* testing on SH-SY5Y cells, it was demonstrated that 34 accurately and selectively identifies FAAH activity within living cells. The intensity of fluorescence emitted by the 34 probe correlates directly with the level of FAAH activity present in the cells. Moreover, the probe can detect variations in FAAH activity when the enzyme is chemically inhibited or genetically suppressed. This approach facilitates the identification of potential FAAH inhibitors and the real-time monitoring of FAAH activity within complex biological systems. Molecular docking analysis was conducted to investigate the binding configuration between neobavaisoflavone and FAAH. The results revealed that neobavaisoflavone can fit within the binding pocket of FAAH, with the hydroxyl group at the 7th position establishing a hydrogen bond with Thr488, a key residue at the entrance of the active site. These findings suggest that neobavaisoflavone holds promise as a novel natural inhibitor of FAAH, warranting further exploration of its biological functions, as depicted in Fig. 25. The employment of probe 34 to assess the impact of neobavaisoflavone on FAAH activity demonstrated that this natural inhibitor displayed significantly stronger inhibitory activity compared to Biochanin A, a previously known natural inhibitor. These results underscore the utility of probe 34 as a dependable instrument for tracking FAAH activity within live cells and for identifying potential enzyme inhibitors, with neobavaisoflavone being a noteworthy example.^98^

**Fig. 26:**
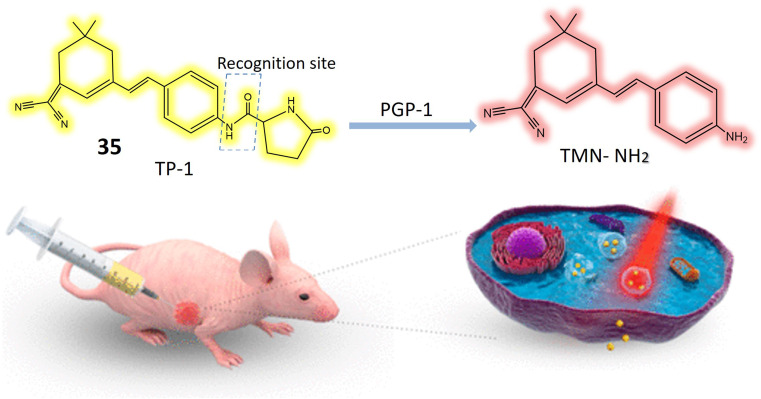
It illustrates that 35 undergoes a fluorescence shift, transitioning from a bright yellow to a red color when exposed to PGP-1. PGP-1 levels tend to rise during inflammation. PGP-1 operates by breaking down the amide bond at a specific recognition site through hydrolysis.

Pyroglutamate aminopeptidase 1 (PGP-1) is notably elevated during inflammatory responses, although the precise correlation between PGP-1 and diseases remains uncertain.^99^ Probe 35 has been meticulously developed for the specific identification of PGP-1, and its exceptional sensitivity and precision contribute to enhanced accuracy in PGP-1 detection. The considerable Stokes shift, with an absorption peak at 400 nm and an emission peak at 564 nm, along with high sensitivity, contributes to the enhanced accuracy in detecting PGP-1. The Stokes shift represents the difference between the absorption and emission wavelengths, and a larger shift, as observed here, reduces spectral overlap and minimizes background interference. This unique property allows for more precise and reliable detection of PGP-1. Additionally, the impressively low detection limit of 0.14 ng mL^−1^ further underscores the sensitivity of the detection method, enabling accurate identification of even minute concentrations of PGP-1. With an increase in PGP-1 concentration, the solution's fluorescence transitions from yellow to a vibrant red color, as illustrated in Fig. 26. The study delves into the interaction between 35 and the enzyme PGP-1. Recognized for its precise substrate recognition capability, enzyme PGP-1 hydrolyzes the amide bond in the chemosensor's structure, resulting in the liberation of the fluorophore and the production of pyroglutamate, which emits vibrant red fluorescence. Utilizing HepG2 cells, RAW264.7 cells, and mice, the investigation explores the link between PGP-1 expression and inflammation. *In vivo* experiments reveal that PGP-1 expression is not only upregulated during inflammation but is even higher in mice afflicted with tumors. Employing a dual-channel ratio signal, near-infrared probe 35 effectively enables real-time monitoring of the enzyme's activity. The probe is also applied to assess HepG2 cells inducing tumors in mice, with the fluorescence intensity of these tumors peaking around 50 minutes. Confocal imaging demonstrates an increase in PGP-1 levels in drug-induced live cells. The results underscore those 35 exhibit exceptionally high sensitivity and rapidity in accurately detecting the enzyme PGP-1. Furthermore, post-interaction with the enzyme, the ratio signal from the near-infrared dual emission channels (644 nm/564 nm) proves more effective in minimizing fluorescence errors. Near-infrared wavelength fluorescent detection methods offer distinct advantages, including low biological toxicity, deep tissue penetration capabilities, and limited interference from background fluorescence originating from biological molecules. These qualities position near-infrared ratiometric fluorescence technique as a valuable tool for studying physiological processes and diseases associated with PGP-1 in mice.^100^

**Fig. 27:**
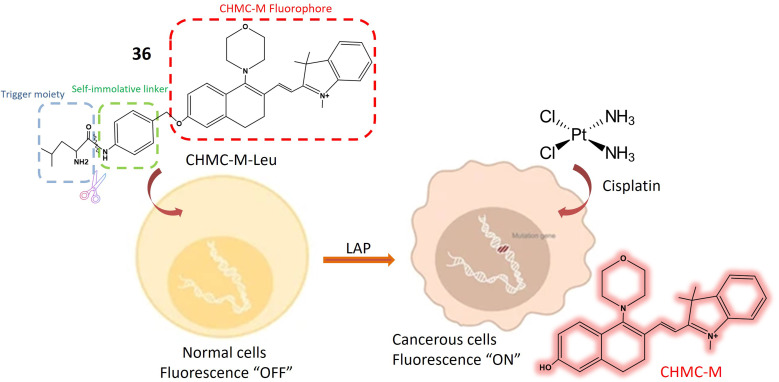
It illustrates that when 36 encounters lap during incubation, the amide bond connecting PABA and l-leucine is broken, releasing *p*-aminobenzyloxyl. Subsequently, this liberated substance undergoes self-immolation, causing the emission of the red fluorescent CHMC-M dye. Additionally, the fluorescence of CHMC-M-Leu intensifies when exposed to cisplatin, mainly because cisplatin activates lap.

Leucine aminopeptidase (LAP) is an enzyme ubiquitously distributed in various tissues and cells, including the liver, kidney, and cells of the small intestine. Its primary function involves cleaving amino acids from the N-terminus of peptide chains or proteins, playing a pivotal role in protein metabolism and amino acid regulation.^101,102^ LAP is frequently overexpressed in cancer cells, serving as a biomarker for cancer diagnosis, prognosis, and treatment response monitoring,^103^ thus contributing to early detection and cancer management.^104^ The 36 fluorescent probe is composed of two main components: l-leucine, acting as the enzyme-active trigger unit, and CHMC-M, functioning as the reporter emitting NIR light. These components are linked by a bridging segment comprising *p*-aminobenzyl alcohol (PABA). Upon incubation with LAP, the amide bond connecting PABA and l-leucine is cleaved, releasing PABA. Subsequent self-immolation of the liberated compound results in the release of the red fluorescent CHMC-M dye, as depicted in Fig. 27. Initially, the probe exhibits low fluorescence at 607 nm when excited at 530 nm, but LAP exposure induces a significant increase in NIR fluorescence at 625 nm (Table 1). The probe underwent *in vitro* and *in vivo* evaluations, showcasing its ability to accurately monitor LAP in real-time within living cells. *In vitro* assessments revealed 36's specific sensitivity to LAP compared to other biological substances, enabling LAP detection in urine and plasma from healthy individuals. The probe offers several advantages, including high water solubility, remarkable stability in living samples, and strong specificity for LAP in the near-infrared range. It effectively penetrates cell membranes, visualizing LAP in small quantities within living cells, and demonstrates significant selectivity over potential interfering substances. To assess its safety, standard MTT assays were conducted in HeLa cells, indicating low toxicity and compatibility with cultured cell lines. Notably, the fluorescence of 36 increases when exposed to cisplatin, as cisplatin activates LAP. LAP's involvement in cisplatin processing results in enhanced fluorescence intensity. Observing LAP activity provides valuable insights into processes underlying cisplatin resistance, offering innovative approaches to combat resistance. Furthermore, the probe enables visualization of small LAP quantities within living cells, aiding in cancer cell identification and progression monitoring.^105^

**Fig. 28:**
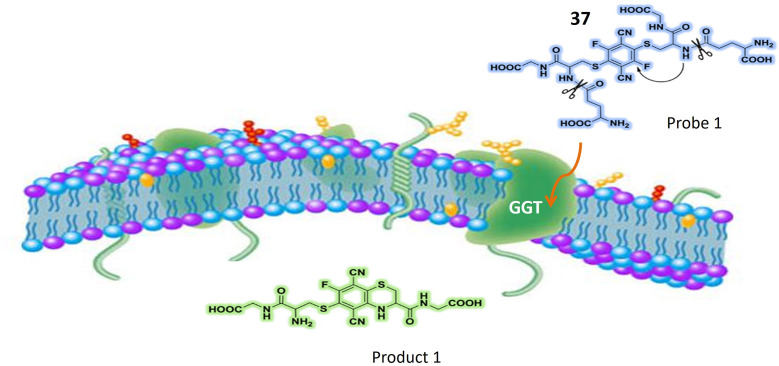
It illustrates that 37 undergoes cyclization after interacting with GGT, it exhibits a noticeable alteration in fluorescence.

Gamma-glutamyl transpeptidase (GGT), also known as c-glutamyl transpeptidase, is an enzyme distributed in various tissues, notably in the liver, biliary tract, and kidney, where it plays a critical role in glutathione metabolism—a potent antioxidant safeguarding cells against oxidative damage.^106,107^ GGT is membrane-bound in these tissues, facilitating the breakdown and transfer of amino acids. Its measurement is crucial for the diagnosis and monitoring of liver diseases, alcohol-related liver damage, and potential cardiovascular risks, serving as a valuable clinical marker guiding medical evaluations and treatments.^108,109^ It functions as a valuable clinical marker, offering insights into diverse facets of overall health and aiding in the guidance of medical evaluations and treatments.^110^ Chemosensor37 is structured around a central terephthalonitrile core with four fluorine atoms attached to aromatic rings, and two glutathione (GSH) molecules linked to the core *via* nucleophilic substitution reactions (Table 1). Upon interaction with GGT, 37 undergoes a distinct reaction leading to a change in its fluorescence emission ratio, detectable and quantifiable. Serving as a ratiometric fluorescent probe, 37 exhibits altered fluorescence color in the presence of GGT. GGT catalysis induces a red shift in the absorption peak from 359 nm to 423 nm, attributed to the cyclization of 37 in response to GGT, as illustrated in Fig. 28. Additionally, the emission wavelength experiences a pronounced red shift, indicating a distinct alteration in fluorescence color compared to 4F-2CN-GSH. Laboratory assessments of 37's ratiometric characteristics involved tracking the fluorescence intensity ratio (FL 494/FL 452) over time in varying GGT activity levels. Results showed a linear increase in the emission ratio from 0.5 to 4.5 over 2 hours. *In vitro* evaluations on HUVECs and HepG-2 cells demonstrated low cytotoxicity and suitability for qualitative assessments of GGT activity within living cells. Assessing selectivity for blood serum applications involved introducing potential interferents, with 37 exhibiting minimal changes in the FL 494/FL 452 ratio except in the presence of GGT, affirming its selectivity. The probe's sensitivity and capacity for quantitative analysis of GGT activity in blood serum were confirmed across normal and abnormal GGT levels encountered in clinical practice. Compared to alternative techniques, probe 37 excels in sensitivity and specificity toward GGT, enabling precise detection even in complex biological environments. Suitable for ratiometric imaging of GGT within living cells, it provides a dynamic perspective of GGT activity. With a low limit of detection (0.042 U L^−1^) and minimal cytotoxicity, 37 holds promise for clinical applications and research. Its capability to detect GGT in cancer cells offers potential for early cancer detection and monitoring of cancer progression, particularly in addressing GGT-related drug resistance.^111^

### 3.7 Chemosensors responsive to glycosidases

Mammalian cell-surface glycans, manifested as glycoconjugates such as glycosphingolipids, glycoproteins, and proteoglycans, play a pivotal role in a diverse array of biological processes through their interactions with proteins.^112,113^ Within the Golgi apparatus and Endoplasmic reticulum, glycosyltransferases and glycosidases collaboratively participate in the intricate process of forming glycan chains on glycoconjugates.^114^ Subsequently, glycosidases present in the lysosomes undertake the crucial responsibility of degrading glycoconjugates.^115^ This interplay between glycosyltransferases and glycosidases is fundamental for the dynamic synthesis and degradation of glycoconjugates. Notably, dysregulation or overexpression of specific glycosidases, such as β-Gal, has been implicated in various diseases, with increased levels of β-Gal associated, for instance, with the metastasis of ovarian cancer.^116,117^ Given the importance of glycosidases in cellular processes and disease pathology, there is a pressing need for robust techniques that facilitate real-time imaging of glycosidases. Such methodologies are indispensable for conducting comprehensive studies on the functional and structural aspects of these biologically significant glycans.^118,119^

**Fig. 29:**
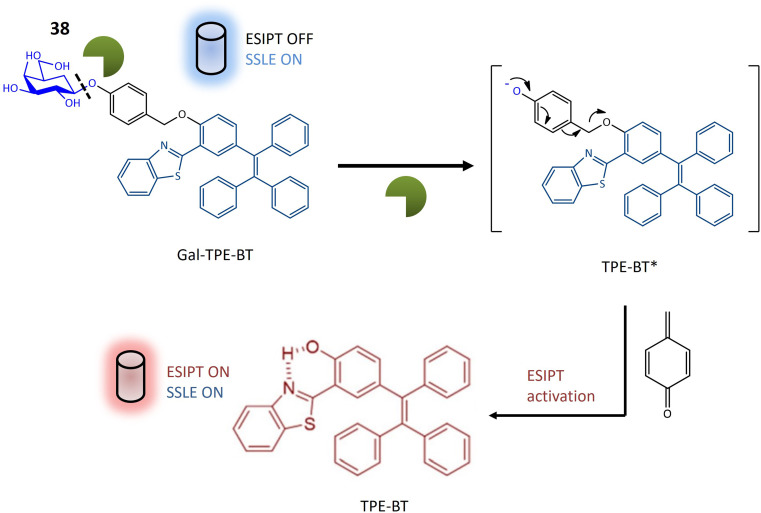
Schematic illustration of the method for detecting β-galactosidase through a combination of excited-state intramolecular proton transfer (ESIPT) and the solid-state luminescence enhancement (SSLE). Probe 38 shows strong fluorescence after interacting with enzyme.

A novel ratiometric “turn-on” fluorescent probe, denoted as 38, has been strategically designed for the precise detection and monitoring of glycosidase activity. The probe operates through a dual mechanism involving solid-state luminescence enhancement (SSLE) and excited-state proton transfer (ESIPT). This dual mechanism response is characterized by a substantial Stokes shift upon glycosidase hydrolysis. Specifically, the probe exhibits standard SSLE characteristics upon glycosylation, while deglycosylation triggers the ESIPT process, resulting in a notable red-shift fluorescence emission of approximately 140 nm (Fig. 29). The molecular structure of 38 was meticulously crafted by incorporating benzothiazole at the ortho position relative to the phenol group on tetraphenyl ethylene (TPE) (Table 1). The conjugation was achieved through the cyclo-addition of 2-aminothiophenol and 2-hydroxy-5-(1,2,2-triphenylethenyl)-benzaldehyde. The presence of excited state intramolecular proton transfer (ESIPT) was induced by the interaction between the phenolic proton and nitrogen atom. To impede ESIPT by eliminating the hydrogen bond donor, galactose was strategically incorporated at the phenolic site. Further enhancing glycosidase sensitivity, a benzyl group was employed as a linker between 38 and glycosidase, resulting in the Gal-TPE-BT probe. The photophysical characteristics of the probe were comprehensively evaluated in both the presence and absence of β-Gal, utilizing an enzyme isolated from *Escherichia coli* as a representative model. The probe exhibited SSLE properties, with minimal fluorescence observed in dimethyl sulfoxide (DMSO) when excited at 360 nm. The gradual addition of water to the solvent system led to a progressive enhancement in fluorescence at its maximum intensity (*λ*_max_ = 470 nm), a distinctive feature of 38. Mass spectroscopic analysis provided concrete evidence of the enzymatic hydrolysis process, revealing a distinct MS peak corresponding to 38 after treatment with β-Gal. The reaction kinetics analysis demonstrated a significant 13.5-fold increase in the (*I*_560_/*I*_440_) ratio after a 300 minutes interaction between the probe and the enzyme. Furthermore, the selectivity of Gal-TPE-BT was rigorously assessed in the presence of unselective enzymes. Notably, exposure to β-glucosidase (β-Glc), an enzyme responsible for glucose breakdown (the C4-epimer of galactose), resulted in minor changes in the fluorescence of the probe. These findings underscore the robust specificity of Gal-TPE-BT for detecting β-galactosidase.^120^

**Fig. 30:**
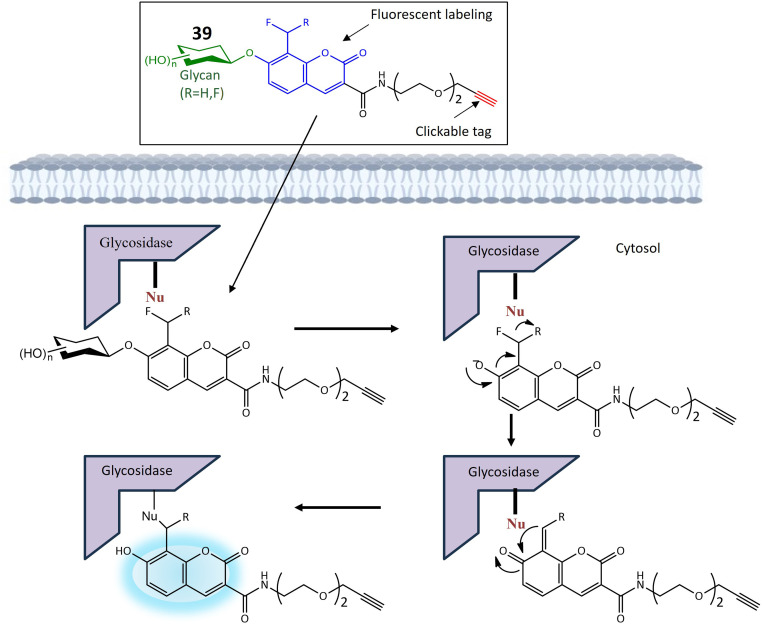
A strategy for employing trifunctional fluorogenic probes 39 to perform fluorescence-based imaging. The figure shows that upon interaction with the targeted glycosidase, the sugar segment of probe 39 undergoes cleavage, leading to the rapid release of fluoride from the fluoromethylated CM group. The resultant quinone methide intermediate subsequently interacts with a nucleophilic site in the side chain of one or more amino acids in the target glycosidase, where it exhibited increased fluorescence.

A novel class of trifunctional fluorogenic probes has been ingeniously designed to serve a dual purpose, facilitating both fluorescence-based cellular imaging and the isolation of cellular glycosidases. These probes comprise three essential components: (1) sugar segment: serving as a substrate for glycosidases. (2) Mono- or difluoromethylated coumarin (CM): used for labeling and visualizing the enzyme through fluorescence. (3) Alkyne tag: facilitating the isolation of the labeled enzyme through affinity chromatography. In its intact form, the probe does not exhibit any fluorescence. However, upon the targeted enzyme (glycosylate) cleaving the sugar segment from the probe, it triggers the release of fluoride from the fluoromethylated CM group. This initiates the formation of a reactive quinone methide intermediate, which subsequently interacts with a nucleophile present in the side chain of one or more amino acids in the targeted glycosidase. This chemical reaction results in the labeling of the enzyme with a fluorescent marker, detectable using fluorescent microscopy. Additionally, the alkyne molecule within the marked glycosidase engages in click chemistry with an azide-linked biotin (N3-biotin), facilitating the retrieval of the labeled glycosidase through affinity chromatography (Fig. 30). To assess the potential of trifunctional fluorogenic probes in detecting glycosidases, both monofluoromethylated probes (39) containing β-Gal, β-Glc, and β-GlcNAc sugar moieties, and difluoromethylated probes (40), were synthesized. The effectiveness of these probes was evaluated by incubating them with target glycosidases, followed by a chemical reaction involving N3-biotin. Results indicated that the 39 probes exhibited higher labeling efficiencies compared to their 40 counterparts. The specificity of the 39 probes toward glycosidases was further investigated. Three enzymes (glycosidases) or *E. coli* cell lysates containing each glycosidase were mixed and incubated with 39. This incubation was conducted both in the presence and absence of glycosidase inhibitors, specific to each case. The analysis of the reaction mixtures using SDS-PAGE demonstrated that the 39 effectively captured the glycosidases. Finally, to assess whether 39 could serve the purpose of fluorescence imaging and separation of glycosidases from mammalian cells, probe 39a, containing β-GlcNAc sugar moieties, was specifically designed to detect the presence of O-GlcNAcase or endogenous glycosidase within mammalian cells. Upon interaction with endogenous glycosidases, the fluorophore moiety formed a stable covalent bond with the enzyme. Consequently, the fluorescence originating from the captured glycosidase remained within the cells even after an extended 2 hours washing period. For the isolation of the cytosolic enzyme (O-GlcNAcase), lysates from HT-29 cells were exposed to 39a, both with and without PUGNAc. The subsequent steps involved a click reaction with N3-biotin, followed by purification using streptavidin. The protein's identity was validated through western blotting in conjunction with the O-GlcNAcase antibody. The strategy employed for the detection and isolation of O-GlcNAcase holds promise for the isolation of other endogenous enzymes.^121^

**Fig. 31:**
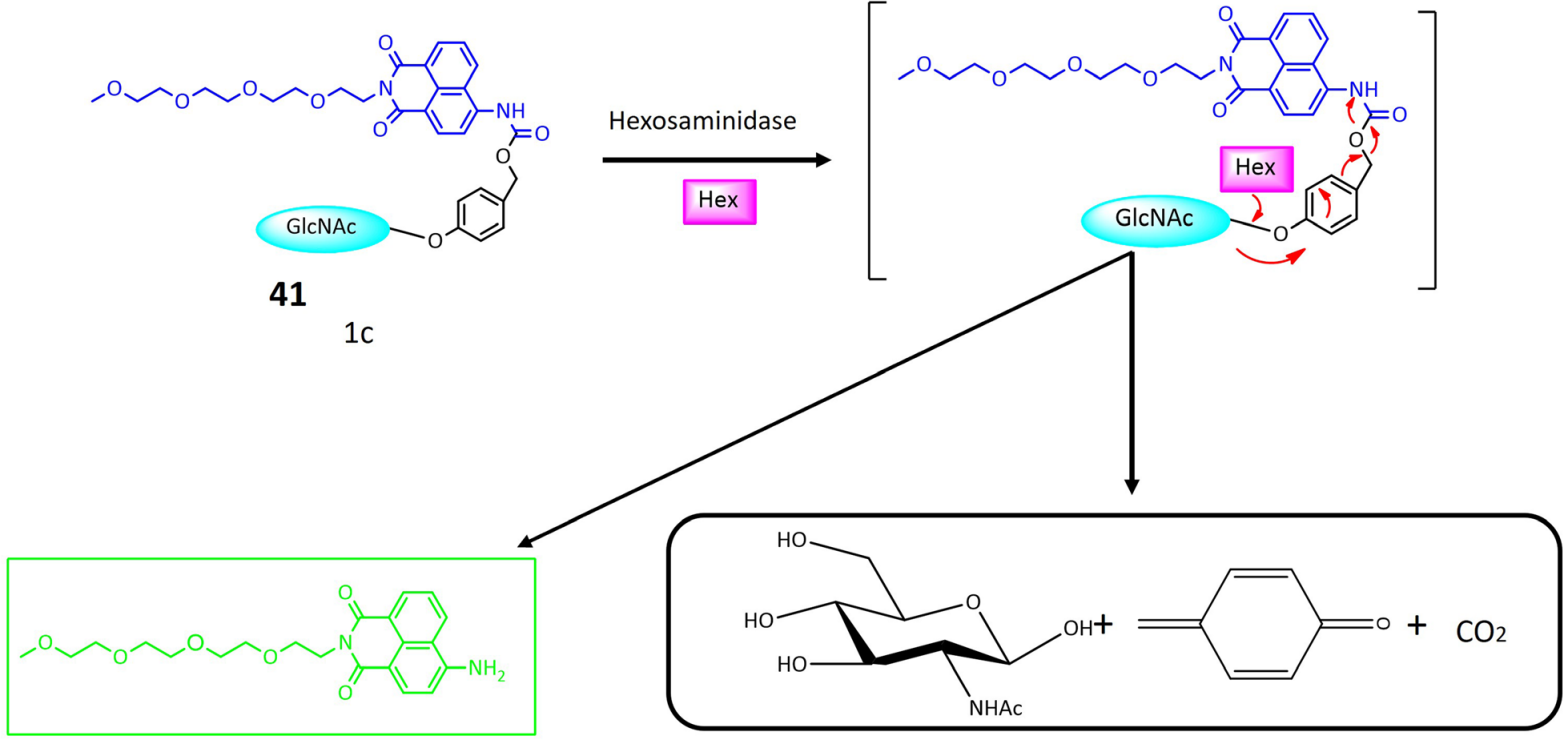
Proposed mechanism of naphthalimide-based activatable fluorescent probe 41 for the assessment of intracellular hexosaminidase activity. Probe 41 exhibited strong fluorescence due to the release of the strong electron-donating amido group after interacting with Hexs.

Hexosaminidases (Hexs) represent a group of enzymes present across various organisms, exerting a widespread influence in the degradation of *N*-acetyl-d hexosamine residues located at the nonreducing end of glycoconjugates. These enzymes play crucial roles in diverse biological processes, including chitin metabolism, defense against pathogenic invasion, modification of *N*-linked glycan structures, egg-sperm identification, regulation of cell division, and involvement in autoimmune diseases in humans. In the pursuit of an efficient tool for rapidly evaluating the inhibition of Hex by small molecules and visualizing Hex activity within cells, ratiometric fluorescent probes based on the naphthalimide structure were meticulously designed. These probes emit two distinct fluorescent signals, leveraging the known intramolecular charge transfer capabilities of naphthalimides containing a 4-amino substituent. The glycosylated naphthalimide-based fluorescence probe 41 was intricately crafted using aminonaphthalimide as the core fluorophore. The amino group at position 4 was modified to include a carbamate moiety, forming an initial structure for an ICT prototype. This structure was further refined by incorporating a *N*-acetyl-β-d-glucosaminide group, responsive to hexosaminidase, as depicted in Fig. 31. Considering the solubility of poly (ethylene glycol) in water, PEG units and a benzene ring were strategically integrated into the fluorescent probe to enhance water solubility and affinity (Table 1). The probe's significant responsiveness is evident through pronounced alterations in both color (shifting from transparent to yellow) and fluorescence (changing from blue to green), attributed to an intramolecular charge transfer process. The potential of probe 41 was further assessed using a human colorectal cancer cell model (SW480) known for its high expression of Hexs. Initial MTT assays demonstrated the non-cytotoxic nature of probe 1c at concentrations up to 80 μM. Subsequent incubation of SW480 cells with the probe at a concentration of 10 μM in PBS buffer at 37 °C for 30 minutes revealed the presence of the probe within SW480 cells through green fluorescence, indicating its interaction with Hexs. These findings underscore the effectiveness of probe 41 for assessing intracellular hexosaminidase activity.^122^

**Fig. 32:**
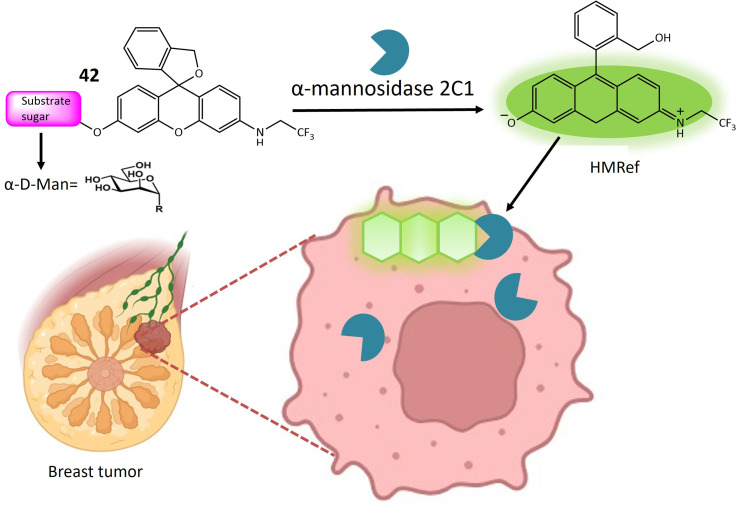
Fluorescence activity of 42 towards α-mannosidase 2C1 in breast tumor cells.

Fluorescence-based techniques for intraoperative cancer detection are regarded as highly promising for evaluating surgical margins. In this context, a series of 12 fluorescent probes, each incorporating distinct sugar moieties such as β-d-Glc, β-d-Gal, β-l-Gal, β-d-Xyl, α-d-Man, β-d-Fuc, α-l-Fuc, β-l-Fuc, α-d-Ara, α-l-Ara, β-d-GlcNAc, and β-d-GalNAc, were meticulously designed. These probes aimed to detect the upregulation of glycosidase activity in breast cancer by integrating various glycosides into the previously described fluorophore, HMRef.^123^ When conjugated with the sugar moiety of an enzyme substrate, HMRef-based probes remain non-fluorescent. However, upon rapid cleavage of the substrate by the target enzyme, they transform into intensely luminous HMRef, enabling the rapid detection of glycosidase activity in living cells. The application of these probes involved the assessment of glycosidase activity in surgically removed specimens from both normal and breast cancer tissues. The tissues, dissected into small millimeter-sized pieces using the freeze-thaw method, were exposed to each of the 12 fluorescent probes. Probes 42 and 43, featuring α-d-Man and β-d-GlcNAc sugar moieties, respectively, exhibited a notable and time-dependent increase in fluorescence specifically within breast cancer tissues, as illustrated in Fig. 32. To confirm the biomarker responsible for fluorescence emission in breast cancer, a diced electrophoresis gel (DEG) assay, combining 2D-gel fluorometry and peptide mass fingerprinting, was conducted. Probe 42 was applied to a ductal carcinoma specimen (IDC), resulting in the observation of a single fluorescent spot. Subsequent peptide mass fingerprinting analysis confirmed the spot's identity as human cytosolic α-mannosidase 2C1, also known as MAN2C1. Immunohistochemical (IHC) staining further validated the elevated expression of MAN2C1 in breast cancer tissue. The α-mannosidase activity was also assessed in phyllodes tumor (PT), intracystic papilloma (ICP), and fibroadenoma (FA), revealing robust fluorescence signals in these breast lesions when exposed to probe 42. Remarkably, the specificity and sensitivity values for distinguishing fibroadenoma from normal breast tissue were both 100%.^124^

**Fig. 33:**
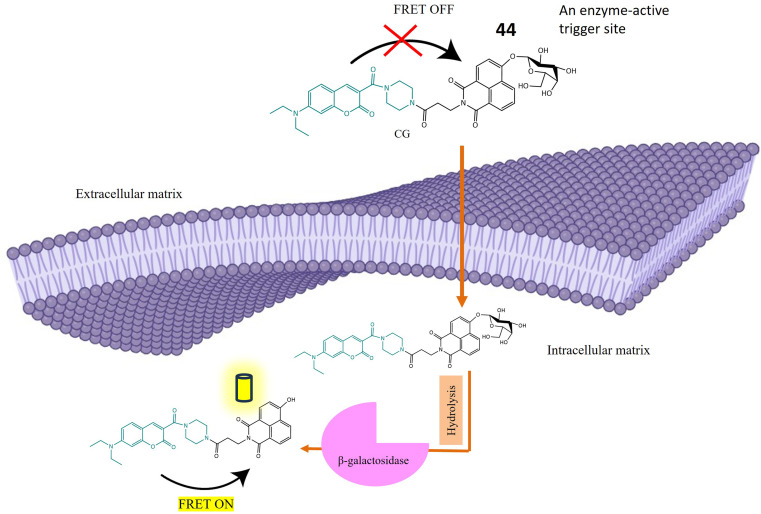
An ultrasensitive fluorescent probe 44 activity based on ICT-FRET mechanism for live cell imaging β-galactosidase. It shows that probe 44 exhibited strong yellow fluorescence upon interaction with β-galactosidase based on ICT-FRET dual processes.

β-Galactosidase, an enzyme integral to the regulation of numerous cellular processes and implicated in the pathogenesis of various diseases, is a subject of investigation using a newly developed probe 44. This probe operates based on the synergistic mechanisms of Fluorescence Resonance Energy Transfer (FRET) and Intramolecular Charge Transfer for the sensitive detection of β-galactosidase activity. In FRET, energy is transferred from a donor (excited dye) to an acceptor (another dye) without the emission of light. The ICT mechanism modulates the electron-donating capability of the probe, thereby adjusting the quantum yield of the fluorescent probes. The concurrent operation of both FRET and ICT mechanisms significantly amplifies the signal generated upon activation by β-galactosidase, as depicted in Fig. 33. The core structure of probe 44 comprises 7-diethylamino coumarin as the donor group and 1,8-naphthalimide as the acceptor moiety (Table 1). In this design, β-d-galactopyranoside is linked to the hydroxy group of the naphthalimide, acting as an enzyme activator when hydrolyzed by β-galactosidase. When the ICT-FRET dual processes are inhibited, 44 exhibits discernible blue fluorescence originating from coumarin in the 500 nm range. Upon hydrolysis by β-galactosidase, 4-hydroxyl-naphthalimide (NH) is produced, leading to a shift in fluorescence intensity towards the red spectrum. Cellular imaging of β-galactosidase activity was conducted using OVCAR3 cells. Initial exposure to an excess of d-galactose (200 μM) for 30 minutes, followed by treatment with 44 (10 μM) for an additional 30 minutes, resulted in noticeable strong blue fluorescence and weak green fluorescence, successfully visualizing β-galactosidase activity within cells. Subsequent assessment of β-galactosidase distribution in both organs and tumors utilized tumor-bearing mouse models. Following the removal of major organs, exposure to 44 (10 μM) produced time-dependent signals, with tumor-bearing organs exhibiting brighter signals at 570 nm compared to other organs.^125^

## 4. Conclusion

In conclusion, the presented body of work underscores the critical role of advanced fluorescent probes in elucidating the intricate dynamics of various enzymes involved in cellular processes, particularly glycosidases. The exploration of diverse probes, each designed with precision and tailored mechanisms, showcases a commitment to advancing our understanding of enzymatic activities within living systems. The elucidation of glycosidase activities, encompassing enzymes such as β-galactosidase, through the strategic integration of cutting-edge technologies like FRET, ICT, and solid-state luminescence enhancement, demonstrates a multidimensional approach to enzyme detection. These methodologies, which leverage the principles of energy transfer and charge modulation, provide not only enhanced sensitivity but also real-time insights into the enzymatic processes occurring within cells. The probes' ability to accurately detect and monitor glycosidase activity in various tissues, including cancerous specimens, highlights their potential applications in clinical settings. These tools offer valuable contributions to cancer diagnosis, with the capability to distinguish between normal and cancerous tissues based on distinct glycosidase expression patterns. Additionally, the probes' selectivity and specificity, as demonstrated in distinguishing different glycosidases and their isoforms, lay the groundwork for further studies in the realm of personalized medicine and targeted therapies. The integration of diverse sugar moieties into the fluorescent probes, coupled with their successful application in breast cancer tissues, exemplifies the versatility and adaptability of these technologies. Moreover, the development of trifunctional fluorogenic probes, capable of not only fluorescence-based cellular imaging but also the isolation of cellular glycosidases, opens avenues for comprehensive studies in enzyme biology and potential therapeutic interventions. In summary, the sophisticated design and successful deployment of these fluorescent probes represent a significant stride in the field of enzymology, offering powerful tools for researchers and clinicians alike. As we navigate the complex landscape of cellular processes, these probes illuminate new pathways for understanding disease mechanisms, enabling early detection, and fostering the development of targeted therapeutic interventions.

We have explored several promising ways for future research and application of advanced fluorescent probes. First, the integration of nanotechnology stands out as a potential catalyst for enhancing the precision and efficiency of fluorescent probes. The utilization of nanoparticle-based probes introduces the prospect of targeted delivery, enabling more localized and effective monitoring of enzymatic activities within specific cellular compartments. Secondly, advancements in probe design are anticipated to play a pivotal role in elevating the capabilities of *in vivo* imaging. This progression holds the potential to revolutionize our comprehension of dynamic cellular activities and significantly enhance diagnostic capabilities, particularly in the early detection of diseases. A third area of exploration involves the synergistic integration of advanced probes with artificial intelligence algorithms. This combination has the capacity to revolutionize data analysis and interpretation, paving the way for more effective disease diagnosis and the formulation of personalized treatment strategies. The marriage of cutting-edge probes and AI algorithms promises to usher in a new era of precision and efficiency in the field. Lastly, the evolving landscape of probe development suggests the possible emergence of theranostic probes. These probes, integrating both diagnostic and therapeutic functionalities, could represent a transformative approach to addressing complex biological processes. The convergence of diagnostic and therapeutic capabilities within a single probe holds great potential for advancing targeted interventions and personalized medicine. As we anticipate these exciting developments, the future of fluorescent probes appears poised for remarkable innovation and transformative applications.

## Conflicts of interest

There are no conflicts to declare.

## Supplementary Material
